# Is ovarian cancer a targetable disease? A systematic review and meta-analysis and genomic data investigation

**DOI:** 10.18632/oncotarget.12633

**Published:** 2016-10-13

**Authors:** Nicoletta Staropoli, Domenico Ciliberto, Silvia Chiellino, Francesca Caglioti, Teresa Del Giudice, Simona Gualtieri, Angela Salvino, Alessandra Strangio, Cirino Botta, Sandro Pignata, Pierfrancesco Tassone, Pierosandro Tagliaferri

**Affiliations:** ^1^ Department of Experimental and Clinical Medicine, Magna Græcia University, Catanzaro, Italy; ^2^ Department of Gynecologic and Urologic Oncology, Fondazione Pascale, National Cancer Institute of Naples, Naples, Italy

**Keywords:** ovarian cancer, targeted therapy, systemic chemotherapy, systematic review, meta-analysis

## Abstract

**Objectives:**

The current gold-standard for the first-line treatment in IIIb/IV stages of epithelial ovarian cancer (EOC) is the combination of carboplatin and paclitaxel *plus* bevacizumab in some countries. In the era of personalized medicine, there is still uncertainty on the impact of several molecularly targeted agents, which have been investigated for the management of this disease. To shed light on the actual role of targeted therapy in EOC, a systematic review and meta-analysis was performed.

**Methods:**

Clinical trials were selected by searching “Pubmed” database and abstracts from major cancer meetings within the time-frame of January 2004-June 2015. The endpoints were survival outcome and response rate (RR). Hazard ratios (HRs) of survival outcomes, with confidence intervals and odds-ratios (ORs) of RR, were extracted from retrieved studies and used for current analysis. Meta-analysis was carried out by random effect model.

**Results:**

30 randomized trials for a total of 10,530 patients were selected and included in the final analysis. A benefit in terms of OS (pooled HR 0.915; 95%CI 0.840-0.997; *p*=0.043), particularly for anti-angiogenetic agents (HR 0.872; 95%CI 0.761-1.000; *p*=0.049), has been demonstrated for targeted therapy. Moreover, a significant advantage in platinum-resistant subgroup in term of PFS (HR 0.755; 95%CI 0.624-0.912; *p*=0.004) was found.

**Conclusions:**

This systematic review and meta-analysis provide the first evidence that targeted therapy is potentially able to translate into improved survival of EOC patients, with a major role played by anti-angiogenetic drugs. The role of target therapy is underlined in the platinum-resistant setting that represents the “pain in the neck” in EOC management.

## BACKGROUND

### Description of epidemiology and clinical management

Epithelial ovarian cancer (EOC) is the leading cause of gynaecologic cancer mortality in developed countries. The overall 5-years survival rate is 30%, due to the absence of validated screening programs which often translates in advanced stage presentation [1]. Surgery is deemed to provide optimal tumour debulking, to assess pathology and to define the FIGO stage [2].

The role of chemotherapy both in adjuvant therapy and first line treatment is well established and carboplatin is still the mainstay of care worldwide [3]. The understanding of EOC biology in term of key events regulating most important signal transduction pathways and angiogenesis has led to the development of novel agents in EOC management [2, 4]. In the last years, 2 clinical trials successfully investigated the role of bevacizumab, an anti-VEGF monoclonal antibody, in the first-line treatment, showing significant advantage in term of progression free survival (PFS) in combination to standard carboplatin and paclitaxel schedule [5, 6]. The selection of second-line treatment takes into account the efficacy of previous therapy, in term of the interval lenght from last platinum administration. On this basis, it is possible to offer platinum re-challenge to patients whose recurrence occurs 12 months after last platinum cycle and a different monotherapy in refractory/resistant platinum patients, whose recurrence occurs within 6 months from last platinum treatment [7–10].

### Hypothesis on disease pathobiology and new classification

Regardless of the anatomical site, several findings indicate that the clinical outcome and prognosis of EOC are highly dependent on molecular and pathological features in which specific mutations (KRAS, PIK3CA, TP53, BRCA1 and BRCA2) are unequally distributed among different subtypes. Indeed, it is presently common thought that EOCs represent a “tree” of distinct pathological entities that share only the anatomic site [11]. On these bases, Shih and Kurman proposed a two-tier model of carcinogenesis, classifying EOC into 2 groups: *Type I* and *Type II*. The *Type I* that arises by precursor lesion and includes neoplasms that are commonly indolent, genetically stable and characterized by poor response to platinum-based chemotherapy; the *Type II*, characterized by de novo lesions, includes high-grade tumors that are usually diagnosed in advanced stages and are genetically unstable: frequently TP53 mutated, carry wild-type RAS genes and often germline or sporadic BRCA1/2 mutations or BRCA1/2 promoter methylation [12]. This last subgroup showed a strong correlation with response to platinum, probably due to early loss of BRCA1/2 and TP53 functions [13]. Moreover, about 50% of sporadic EOC display defects in the DNA repair homologous recombination (HR) pathway with subsequent inability to repair double-strand breaks induced by platinum compounds, as demonstrated in experimental *in vitro* and *in vivo* models [14–17]. Often, these patients report increased reliance on the poly (ADP-ribose) polymerase (PARP) single-strand repair pathway, although this evidence is recognized mostly in BRCA1/2 germline mutations carriers.

In a recent report from Cancer Genome Atlas (TCGA) Research Network 489 cases of high grade serous papillary EOC (HGS-OvCa) were analyzed by micro-arrays mRNA and miRNA profiling and genome sequencing [14]. This work provided the opportunity to identify 4 subtypes based on the expression of marker genes: “Differentiated”, “Immunoreactive”, “Mesenchymal” and “Proliferative” with a potential prognostic and predictive role [18]. To validate this classification several retrospective sub-analyses on ICON7 trial demonstrated that it is possible to correlate a different outcome between the arms by gene expression and the use of biomarkers [6, 19–21].

### Role of inflammation, angiogenesis and molecular pathways involved

Several studies investigated the role of inflammation, immune system and angiogenesis driving the idea that synthesis of cytokines, such as TNF-α, IL-1β, IL-6, PGE-2 and vascular endothelial growth factor (VEGF) by cells from the microenvironment, promotes the onset and development of EOC [22]. A possible explanation of the central role of inflammation can be related to the inflammatory microenvironment that releases IL-6, whose levels are linked to poor prognosis, disease progression, residual disease after debulking surgery, ascites or anemia [23, 24]. Indeed IL-6 seems to play a key role in determining platinum-resistance inducing HIF-1 and STAT-3 expression/activity that promotes VEGF overexpression [25, 26]. In turn VEGF supports ascites production, by increasing peritoneal permeability and immune suppression, by impairing dendritic cells maturation and Th1 response [27].

Although angiogenesis seems the major pathway involved in pathogenesis and progression of EOC, the epidermal growth factor receptor (EGFR)-family plays an important role in different malignancies and EGFR overexpression is correlated to decreased survival in EOC [28]. About 30-98% of EOC present overexpression in one of these pathways. In particular, EGFR pathway seems to have a central role in cell proliferation, migration and invasion through the activation of several signalling pathways, such as RAS-RAF-mitogen-activated protein kinase pathway (RAS/RAF/MAPK pathway) that is able to determine a constitutive activation of STAT-3 and STAT-5 and the phosphatidylinositol 3-kinase pathway (PI3K) [29].

The aim of this work is to provide answer to the basic question if available literature actually supports the concept that molecular targeted agents indeed represent valuable tools for the treatment of EOC. In this light, we attempted to identify the relevance of single targeted-pathway in molecularly unselected EOC patients and in several subgroups recognized by clinical criteria.

## RESULTS

### Study selection and characteristics

**Figure 1:**
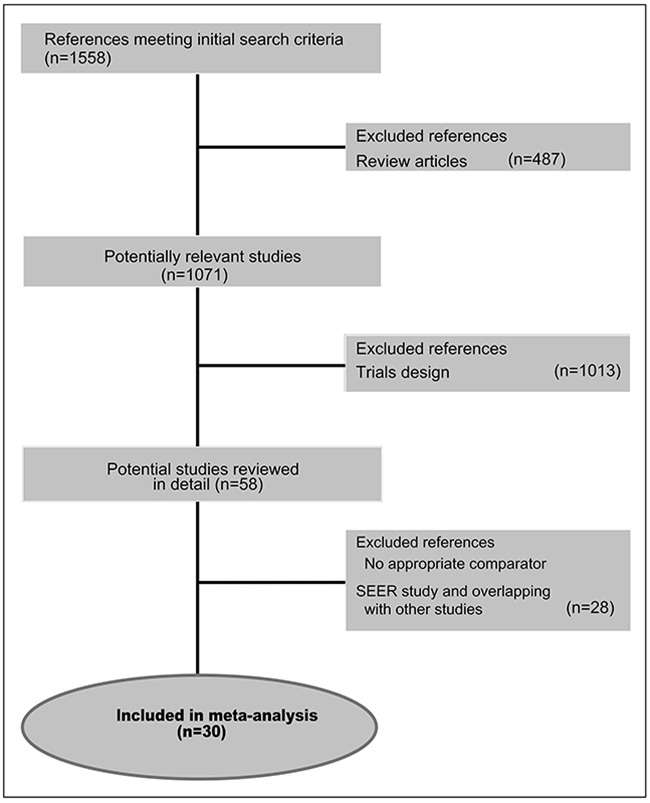
PRISMA chart showing the trial exclusion and inclusion process in the meta-analysis

The PRISMA chart related to RCTs selection and search strategy is described in Figure 1. In the considered time-frame (2004-2015), 1558 studies were identified as full papers or meeting abstracts, while 1500 studies were initially excluded because reviews and/or for trial design. Thus, we examined in detail the remaining 58 trials. Among them, 28 trials were excluded because selection criteria were not met [30]. 30 trials for a total of 10530 patients were selected and included in the final analysis [5, 6, 10, 31–57].

In particular, 2 trials, both designed for multiple arms comparison, were analyzed for single comparison while 9 trials provided a primary treatment followed by a maintenance phase. At least one data-comparison in terms of survival outcome or RR was reported in all selected RCTs, which were therefore deemed eligible for the end-point analysis. Summarizing the 30 trials included in final analyses: 19 were eligible for OS analysis (among them, we underlined, that: 10 were included in anti-angiogenetic analysis; 3 studies were included in anti-EGFR analysis; 3 studies were included in anti-PARP/DNA repair analysis; 3 trials were included in miscellaneous analysis); 27 were eligible for PFS analysis (among them, we underlined, that: 13 were included in anti-angiogenetic analysis; 4 studies were included in anti-EGFR analysis; 2 studies were included in anti-PARP/DNA repair; 8 trials were included in miscellaneous analysis), and 22 were evaluable for RR analysis (among them, we underlined, that: 10 were included in anti-angiogenic analysis; 3 studies were included in anti-EGFR analysis; 3 studies were included in anti-PARP/DNA repair; 6 trials were included in miscellaneous analysis).

### OS analyses

**Figure 2:**
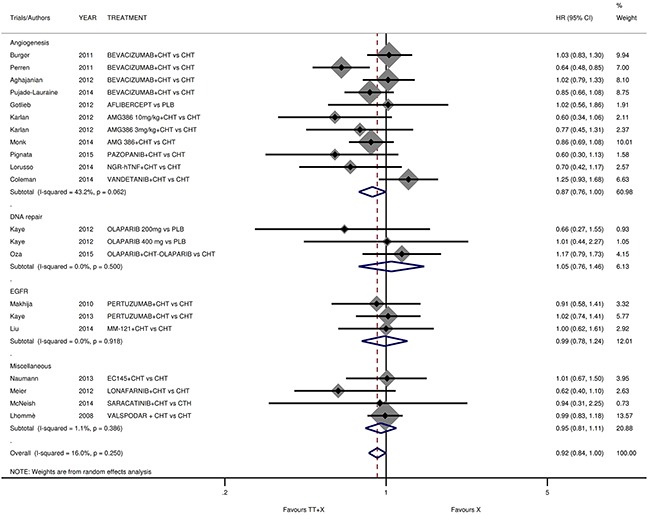
Comparison of OS according to involved pathway Abbreviation: overall survival, OS; hazard ratio, HR; TT: target therapy; X: conventional therapy.

Eleven trials were excluded from OS analysis because of missing data. Our OS analysis showed that targeted therapy *plus* conventional therapy produced a statistically significant, but marginal benefit in EOC patients compared to conventional therapy alone (pooled HR 0.915; 95%CI 0.840-0.997; *p*=0.043; Figure 2). We reported a subgroup analysis on target-therapy pathway. In particular, a significant benefit for anti-angiogenetic agents only, in terms of OS (HR 0.872; 95%CI 0.761-1.000; *p*=0.049), was demonstrated (Supplementary data, Supplementary Figure S1). No statistically significant difference was found for other pathways. We performed a single meta-analysis considering 3 subgroups: platinum-status, line of treatment and maintenance without evidence of significant differences in the subgroups for each analysis (Supplementary data, Supplementary Figures S2–S4).

### PFS analyses

**Figure 3:**
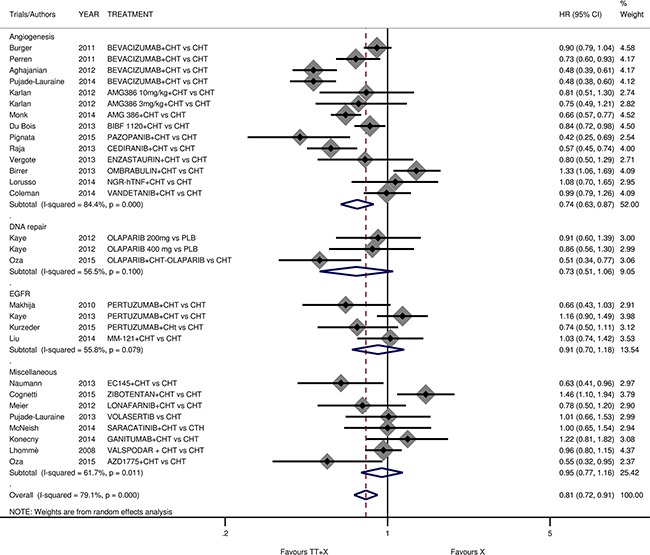
Comparison of PFS according to involved pathway Abbreviation: progression free survival, PFS.

Three trials were excluded from PFS analysis because of missing data. By our PFS analysis targeted therapy-based treatment demonstrated a significant benefit compared to a conventional treatment (pooled HR 0.807; 95%CI 0.717-0.907; *p*<0.001; Figure 3). In more detail, we showed a significant benefit for anti-angiogenetic agents only, in terms of PFS (HR 0.740; 95%CI 0.628-0.872; *p*<0.001). Moreover, we reported a significant advantage in subgroup analysis in relation to the line of treatment (HR 0.792 in second line *versus* 0.860 in first line; *p*=0.004 *versus* 0.006, respectively) (Supplementary data, Supplementary Figures S5). In subgroup analysis for platinum-sensitivity, we reported an interesting and statistically significant benefit in platinum-resistant patients only (HR 0.755; 95%CI 0.624-0.912; *p*=0.004) (Supplementary data, Supplementary Figures S6). Finally, in subgroup with a maintenance (post-combination) phase, we reported a limited but statistically significant benefit in studies with or without maintenance (HR 0.709 in maintenance group *versus* 0.850 in no maintenance group; *p*=0.002 *versus* 0.021, respectively) (Supplementary data, Supplementary Figures S7).

### RR analyses

We excluded from this analysis 8 trials because missing data in terms of RR. No advantage was reported in RR analysis, (OR for RR 1.235; 95%CI 0.970-1.571; *p*=0.087; Supplementary data, Supplementary Figure S8). In the anti-angiogenetic drugs analysis, we reported a significant improvement in term of RR (OR for RR 1.491; 95%CI 1.042-2.134; *p*=0.029). No differences were reported in our subgroup analyses.

### Risk of bias in individual studies

**Figure 4:**
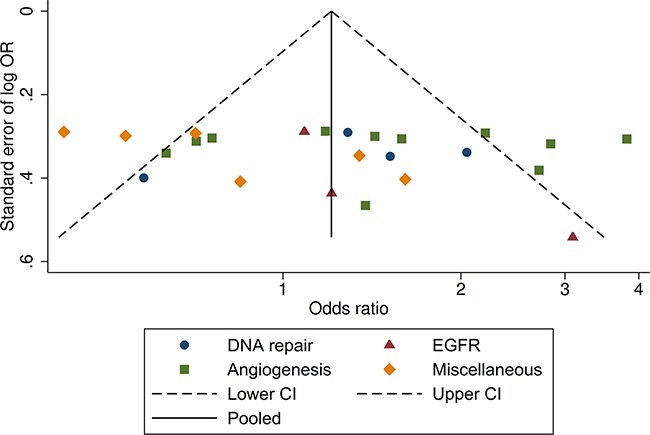
Funnel plot (Begg's test) assessing publication bias

Begg's funnel plot and visual inspection showed a balanced evidence of publication bias (p=0.386) (Figure 4).

## DISCUSSION

This meta-analysis of 30 RCTs, containing 10,530 patients, compares regimens including targeted-agents *versus* systemic conventional treatments, stratified for each molecular pathway. While the aim of this work was the analysis of each pathway, we found a survival benefit for targeted-therapy in its whole (OS: pooled HR 0.91; PFS: pooled HR 0.80). Moreover, a benefit of targeted-agents also in a subgroup analysis due to the effect of anti-angiogenetic agents on survival endpoints was observed (HR 0.87; HR 0.74 for OS and PFS respectively). Importantly, a significant PFS gain in the platinum-resistant patients was found (HR 0.75). To validate the findings here described, we performed sensitivity analyses on single involved pathway confirming pooled results previously reported.

Taking into account these findings, we may afford some possible explanations. First, our results underlined a significant advantage of anti-angiogenic therapy on all endpoints; RCTs investigating various anti-angiogenic agents in the treatment of EOC individually reported PFS benefit without OS advantage, aside from ICON6, for which a significant OS difference was reported [4, 58–60]. The efficacy of anti-angiogenetic therapy indicates that ovarian cancer is highly dependent on angiogenesis in advanced stage of disease.

Furthermore, we conducted an exploratory analysis comparing anti-angiogenetic TKIs (tyrosine-kinase inhibitors) and bevacizumab benefits, but we failed to demonstrate a difference in term of survival endpoints. These findings indicate the occurrence of a class effect at meta-analytic evaluation and provide proof of concept for novel biomarker driven investigation. In our opinion the most interesting finding is the significant PFS benefit in platinum-resistant setting that underscored the importance of this class of drugs, in particular anti-angiogenic agents, in a subgroup with poor prognosis, considered resistant to conventional systemic treatment or surgery [8, 61]. As mainly reported in the AURELIA trial, we show that the addition of anti-angiogenetic agents to standard chemotherapy produces significant benefits, particularly in combination with paclitaxel [10]. A possible explanation can rely on the thought that paclitaxel, administered in weekly schedule, has an anti-angiogenetic mechanism by itself [62, 63]. However, the results from MITO11 trial, that investigated the combination of weekly paclitaxel with pazopanib, did not produce comparable findings [35]. It is possible to speculate that bevacizumab is able to produce in EOC a benefit which might reside not only in the anti-angiogenic activity but also in the immuno-modulating and microenvironment-related effect [64]. It might be also hypothesized that bevacizumab could represent the optimal management for patients with a “mesenchymal disease”, identified by the previously described new molecular classification. Conversely, it is possible that in “immune-reactive” disease new promising agents, such as anti-PD1 and anti-PDL1, could represent the optimal choice for the remarkable immune-system- and inflammation-dependency [65]. Unfortunately, we could not analyze this pathway because trials are still ongoing. Moreover, taking into account this new knowledge, an interesting research area relies on the possible role of microRNAs as therapeutic target [66, 67]. Furthermore, the entity of benefit of targeted-agents is marginal in all reported subgroups examined, probably due to the absence of *a priori* selection of patients.

To date, several confirmatory trials, still ongoing, are designed at the aim of identifying potential predictive biomarkers in order to optimize the use of targeted-therapy [68].

In order to support our findings, we performed an exploratory analysis, using web-available datasets, at the aim to evaluate the expression of major potential biomarkers. In particular we accessed retrievable data from TCGA through CAN-EVOLVE portal (http://www.canevolve.org/AnalysisResults/AnalysisResults.html). By Fisher test analysis, we recognized high expression of VEGFA in EOC. This analysis was validated with Mann-Whitney test in a free dataset (GSE14407) available in web, in which we confirmed that high expression of VEGFA, IL1b and CD31 genes were indeed associated with disease, underlining a potential role of inflammation and angiogenesis as driver pathways in EOC. In our opinion, these findings appear in line with the class-effect of anti-angiogenetic drugs. However, IL-6 and IL-8 did not show significant difference between normal and pathologic tissues in all datasets. Moreover, we observed a correlation between EOC and overexpression of ERBB2 that is generally reported in < 20% of EOC patients (Supplementary data, Supplementary Figures S9–S10). However, in our subgroup analysis on this pathway, we reported the absence of significant benefit. This finding could be explained taking into account that patients where not stratified on this biomarker. The role of maintenance is not established for the management of EOC. Indeed, several trials reported an improvement of PFS not confirmed in overall survival despite this approach. To date the major evidence for maintenance setting in EOC was limited to post-second-line treatment and for a particular subgroup of platinum sensitive patients with a recognized BRCA mutation that does not represent the aim of this manuscript.

Regarding the maintenance phase, in our analysis, we investigated only studies in which the targeted-therapy combined to chemotherapy was also offered in this phase.

Our results did not demonstrate a clear superiority of this approach on survival outcome, but lay the groundwork for the assessment of the studies that evaluate only targeted-therapy maintenance phase independently of previous treatment (e.g. olaparib, pazopanib, erlotinib). These studies, as specified in the methods of this work, were beyond the scope of this meta-analysis.

Regarding the response to targeted-drugs, it is known that RECIST criteria are not the most appropriate approach for measuring and monitoring target lesions in biological therapy. This consideration could justify the lack of significant benefit of targeted-therapy on RR endpoint.

Toxicity analyses are not shown in detail, but we found that diarrhea represents the only adverse event consistently reported in the experimental arms for all pathways, particularly for trials investigating TKIs. Hypertension and vascular events were observed with anti-angiogenetic agents, while skin toxicities were reported with anti-EGFR agents according to Literature data. It would be interesting to correlate toxicity with the possible efficacy of these drugs by the use of novel pharmacogenomics platforms, such as DMET [69]. This powerful approach is indeed suitable for biomarker identification for personalized EOC treatment.

Nevertheless, an important limitation of this meta-analysis is that several investigated drugs did not represent a real option in the clinical practice, except for bevacizumab. Moreover, this work has been performed on Literature-derived data that do not allowed to retrieve all data related to all subgroups, and possibility to aggregate all the data of each single study for all selected endpoints.

Although PFS represent primary endpoint of several involved trials, this outcome could not represent at present an adequate surrogate endpoint for OS. Thus, our results in terms of overall survival must be interpreted in accordance with primary endpoint and not with an inferencial intent.

In the light of our results demonstrating OS benefit of targeted-therapy as a class-effect, it must be underlined that new clinical trials on pre-defined predictive biomarkers are eagerly awaited for *a priori* selection of patients in order to maximize the efficacy of these drugs and drastically reduce the costs of these treatments according to the health technology assessment. A possible new investigative approach could be provided by new trial design. Indeed this important “knowledge gap” represents a major limitation for development of a personalized therapeutic algorithm in the precision medicine era [70]. However, irrespective of the limitations of this work, we can conclude that both the good tolerability profile and the survival benefit indicate that targeted-therapy is potentially able to translate into improved survival of EOC patients, particularly in platinum-resistant setting that represent the “pain in the neck” of the EOC management.

## MATERIALS AND METHODS

### Study design

In order to evaluate the role of targeted therapy-based schedules compared to conventional therapies in the management of EOC, we performed a systematic review and meta-analysis of all published prospective and RCTs designed on EOC in all treatment lines. Overall survival (OS), PFS and response rate (RR) represent the predefined endpoints.

### Searching

Bibliographic research was conducted by PubMed, Embase, and the Central Registry of Controlled Trials of the Cochrane Library, major meeting proceeding databases. The selected time frame referred was between January 2004 and June 2015 because, at our knowledge no modern targeted therapies were evaluated prior to that time. In order to reduce or minimize the risk of selection and information bias, only prospective and RCTs were evaluated in this analysis [71, 72]. The identified key-words are: “ovarian”, “ovary”, “tumor”, “cancer”, “advanced”, “metastatic”, “therapy”, “targeted”, “prospective”, and “randomized” in different combinations: i.e. “epithelial ovarian cancer, targeted therapy”. The ‘related articles’ function and references retrieved from articles were used to perform the search of all related studies, abstracts and citations.

### Selection

In Table 1 are described all characteristics reported by patients enrolled in this work.

**Table 1:** Main characteristics of the randomised trials included in the meta-analysis

TRIALS (first author)	YEAR	TREATMENT	TARGETED PATHWAY	Platinum status	PATI- ENTS	RR control arm	RR experimental arm	OS	PFS
						(%)	(%)	HR	HR
**Burger [9]**	2011	BEVACIZUMAB+CHT vs CHT	angiogenesis		1873	NR	NR	1.03	0.9
**Perren [10]**	2011	BEVACIZUMAB+CHT vs CHT	angiogenesis		1528	48	67	0.64	0.73
**Pujade-Lauraine [15]**	2014	BEVACIZUMAB+CHT vs CHT	angiogenesis	resistant	361	12	27	0.85	0.48
**Aghajanian [50]**	2012	BEVACIZUMAB+CHT vs CHT	angiogenesis	sensitive	484	57	79	1.02	0.48
**Gotlieb [51]**	2012	AFLIBERCEPT vs PLB	angiogenesis	resistant	55	NR	NR	1.02	NR
**Karlan [52]**	2012	AMG386 10mg/kg+CHT vs CHT	angiogenesis	sens/resis	108	27	37	0.6	0.81
**Karlan [52]**	2012	AMG386 3mg/kg+CHT vs CHT	angiogenesis	sens/resis	108	27	19	0.77	0.75
**Monk [53]**	2014	AMG 386+CHT vs CHT	angiogenesis	resistant	919	30	38	0.86	0.66
**Pignata [54]**	2015	PAZOPANIB+CHT vs CHT	angiogenesis	resistant	74	25	56	0.6	0.42
**Vergote [55]**	2013	ENZASTAURIN+CHT vs CHT	angiogenesis		142	39	43	NR	0.8
**Raja [62]**	2013	CEDIRANIB+CHT vs CHT	angiogenesis	sensitive	456	NR	NR	NR	0.57
**Birrer [68]**	2013	OMBRABULIN+CHT vs CHT	angiogenesis	sensitive	154	71	65	NR	1.33
**Lorusso [67]**	2014	NGR-hTNF+CHT vs CHT	angiogenesis	resistant	109	NR	NR	0.7	1.08
**Hainsworth [70]**	2015	SORAFENIB+CHT vs CHT	angiogenesis		85	74	67	NR	NR
**Coleman [74]**	2014	VANDETANIB+CHT vs CHT	angiogenesis	resistant	131	9	12	1.25	0.99
**Du Bois [75]**	2013	BIBF 1120+CHT vs CHT	angiogenesis		1366	NR	NR	NR	0.84
**Kaye [56]**	2012	OLAPARIB 200mg vs PLB	DNA repair	sens/resis	65	18	25	0.66	0.91
**Kaye [56]**	2012	OLAPARIB 400 mg vs PLB	DNA repair	sens/resis	65	18	31	1.01	0.86
**Oza [63]**	2015	OLAPARIB+CHT-OLAPARIB vs CHT	DNA repair	sensitive	162	58	64	1.17	0.51
**Kummar [64]**	2015	VELIPARIB+CHT vs CHT	DNA repair	resistant	74	19	12	NR	NR
**Makhija [57]**	2010	PERTUZUMAB+CHT vs CHT	EGFR	resistant	130	5	14	0.91	0.66
**Kaye [58]**	2013	PERTUZUMAB+CHT vs CHT	EGFR	sensitive	149	59	61	1.02	1.16
**Kurzeder [65]**	2015	PERTUZUMAB+CHt vs CHT	EGFR	resistant	154	NR	NR	NR	0.74
**Liu [66]**	2014	MM-121+CHT vs CHT	EGFR	resistant	223	11	13	1	1.027
**Meier [59]**	2012	LONAFARNIB+CHT vs CHT	miscellaneous		105	NR	NR	0.62	0.78
**Naumann [60]**	2013	EC145+CHT vs CHT	miscellaneous	resistant	149	12	18	1.01	0.63
**Cognetti [61]**	2015	ZIBOTENTAN+CHT vs CHT	miscellaneous	sensitive	120	59	38	NR	1.46
**Pujade-Lauraine [69]**	2013	VOLASERTIB vs CHT	miscellaneous	resistant	109	15	13	NR	1.01
**Konecny [71]**	2014	GANITUMAB+CHT vs CHT	miscellaneous		170	NR	NR	NR	1.22
**Lhommè [72]**	2008	VALSPODAR + CHT vs CHT	miscellaneous		762	42	34	0.99	0.96
**McNeish [73]**	2014	SARACATINIB+CHT vs CTH	miscellaneous	resistant	107	43	29	0.94	1
**Oza [76]**	2015	AZD1775+CHT vs CHT	miscellaneous	sensitive	121	76	81		0.55

Abbreviations: overall survival, OS; progression free survival, PFS; hazard ratio, HR; TT: target therapy; ST: standard therapy; chemotherapy, CHT; best supportive care, BSC; not reported, NR.

### Inclusion criteria

Following are reported the inclusion criteria: Patients with diagnosis of EOC; Prospective and randomized clinical trials (RCTs), with or without blinding; abstracts or unpublished data if sufficient information on study design, characteristics of participants, interventions, and outcomes were available. We identified as experimental arm the targeted therapy-based schedule while the control arm was a conventional schedule for disease stage.

### Exclusion criteria

Non-comparative studies; non-prospective studies; non-comparable end-points; way of chemotherapy or targeted agents administration different from systemic, or oral (e.g. Intra-arterial or intra-peritoneal infusion). We excluded all trials focused only on maintenance phase of treatment.

### Data extraction

In order to select homogeneous studies, 2 investigators (N.S. and D.C.) examined each trial, independently [73]. Any discrepancy was resolved by an arbiter (P.T.). From selected trials identified, the following variables were evaluated and efficacy results were extracted: first author, number of patients enrolled, year of publication, treatment schedule, involved pathway, a maintenance phase after combination treatment with the same targeted-agent, and so on. Efficacy endpoints previous specified (OS, PFS, RR) were analyzed. Data extraction was conducted according to the PRISMA statement.

### Quality assessment

The quality assessment of selected studies was performed according to the Cochrane reviewers' handbook for five requirements: method of randomization, allocation concealment, blindness, withdrawal/dropout, and adequacy of follow-up [74]. Twenty trials were scored A (low risk of bias), 9 trials was scored B (intermediate risk of bias), and 1 trial was scored C (high risk of bias) (Table 2).

**Table 2:** Quality assessment

Included studies	Year	Method of randomization	Allocation concealment	Blind	Withdrawal and dropout	Baseline	Quality level^a^
**Burger [9]**	2011	centralized	central office	yes	Detailed criteria	identical baseline	A
**Perren [10]**	2011	centralized	central office	no	Detailed criteria	identical baseline	A
**Pujade-Lauraine [15]**	2014	No detailed	No detailed	no	Detailed criteria	identical baseline	B
**Aghajanian [50]**	2012	centralized	central office	yes	Detailed criteria	identical baseline	A
**Gotlieb [51]**	2012	centralized	central office	yes	Detailed criteria	identical baseline	A
**Karlan [52]**	2012	centralized	central office	yes	Detailed criteria	identical baseline	A
**Monk [53]**	2014	centralized	central office	yes	Detailed criteria	identical baseline	A
**Pignata [54]**	2015	centralized	central office	no	Detailed criteria	identical baseline	A
**Vergote [55]**	2013	centralized	central office	yes	Detailed criteria	identical baseline	A
**Kaye [56]**	2012	centralized	central office	no	Detailed criteria	identical baseline	A
**Makhija [57]**	2010	centralized	central office	yes	Detailed criteria	identical baseline	A
**Kaye [58]**	2013	No detailed	no detailed	no	Detailed criteria	identical baseline	B
**Meier [59]**	2012	centralized	central office	no	Detailed criteria	identical baseline	A
**Naumann [60]**	2013	centralized	central office	no	Detailed criteria	identical baseline	A
**Cognetti [61]**	2013	centralized	central office	yes	Detailed criteria	identical baseline	A
**Raja [62]**	2013	centralized	central office	yes	Detailed criteria	identical baseline	A
**Oza [63]**	2015	centralized	central office	no	Detailed criteria	identical baseline	A
**Kummar [64]**	2015	No detailed	no detailed	no	no detailed	identical baseline	C
**Kurzeder [65]**	2015	centralized	central office	yes	Detailed criteria	identical baseline	A
**Liu [66]**	2014	No detailed	no detailed	no	Detailed criteria	identical baseline	B
**Lorusso [67]**	2014	No detailed	no detailed	no	Detailed criteria	identical baseline	B
**Birrer [68]**	2013	centralized	central office	yes	Detailed criteria	identical baseline	A
**Pujade-Lauraine [69]**	2013	No detailed	no detailed	no	Detailed criteria	identical baseline	B
**Hainsworth [70]**	2015	No detailed	no detailed	no	Detailed criteria	identical baseline	B
**Konecny [71]**	2014	centralized	central office	yes	Detailed criteria	identical baseline	A
**Lhommè [72]**	2008	No detailed	no detailed	no	Detailed criteria	identical baseline	B
**McNeish [73]**	2014	centralized	central office	no	Detailed criteria	identical baseline	A
**Coleman [74]**	2014	No detailed	no detailed	no	Detailed criteria	identical baseline	B
**Du Bois [75]**	2013	centralized	central office	yes	Detailed criteria	identical baseline	A
**Oza [76]**	2015	No detailed	no detailed	no	Detailed criteria	identical baseline	B

a See Methods for definition

### Quantitative data synthesis

In order to evaluate the effects of the targeted-based treatments [chemotherapy +/− biologicals] in EOC management, we carried out this meta-analysis on pre-specified end-points [75]. We extracted survival data as hazard ratios (HRs) of OS, and PFS with relative confidence intervals (95%CI). The interaction between survival and experimental treatment was obtained by each study from the HRs logarithm. Method for dichotomous data (odds ratio assessment; 95%CI) was used for calculating the overall effect of combined treatments on RR. Cochrane's Q-test and I^2^ statistics were used to assess heterogeneity between studies and the random-effects model was used for the analysis taking into account the intent of comparing trials based on drugs with different mechanisms of action. Pooled data analysis was performed according to the DerSimonian and Laird test [76]. The presence of publication bias was investigated through Begg's test by visual inspection of funnel plots [77]. A two-tailed *p* value equal or lower than 0.05 was considered statistically significant. All the statistical analyses were performed by using STATA SE v. 14.1 (STATA_Corporation, Texas, USA) [78].

### Genomic dataset analysis

We examined available Web-datasets for each gene involved in the pathways reported in our meta-analysis in order to identify the possible correlations between targeted agents and pathway related gene-expression. In particular, we carried out 2 different analyses: regarding TCGA dataset (584 samples of EOC) accessed through CANEVOLVE portal (http://www.canevolve.org/AnalysisResults/AnalysisResults.html), the analyses were conducted by Fisher test; concerningGSE14407 free web-dataset, including 24 samples (12 normal and 12 pathological tissues), the analyses were conducted by Mann-Whitney test to confirm association with disease. A two-tailed *p value* equal or lower than 0.05 was considered statistically significant. All the statistical analyses were performed using Graphpad Prism v.6.

### Contributors

Nicoletta Staropoli, Domenico Ciliberto, Alessandra Strangio and Simona Gualtieri performed the systematic review and the meta-analysis of pooled data, Francesca Caglioti, Silvia Chiellino and Teresa Del Giudice searched the WEB databases and Cirino Botta took care of bioinformatics. Pierosandro Tagliaferri and Pierfrancesco Tassone supervised the work and together to Nicoletta Staropoli and Domenico Ciliberto wrote the paper. Angela Salvino and Sandro Pignata provided expert opinion and participated to all steps of study completion.

## SUPPLEMENTARY FIGURES


