# Supplementary material for: Is ovarian cancer a targetable disease? A systematic review and meta-analysis and genomic data investigation
Source: Oncotarget. 2016 Oct 13;7(50):82741–56. doi: 10.18632/oncotarget.12633 (PMC5347729; doi:10.18632/oncotarget.12633)
Supplement: Supplementary file 1 [file oncotarget-07-82741-s001.pdf]

Is ovarian cancer a targetable disease? A systematic review and meta-analysis and genomic data investigation

SUPPLEMENTARY FIGURES

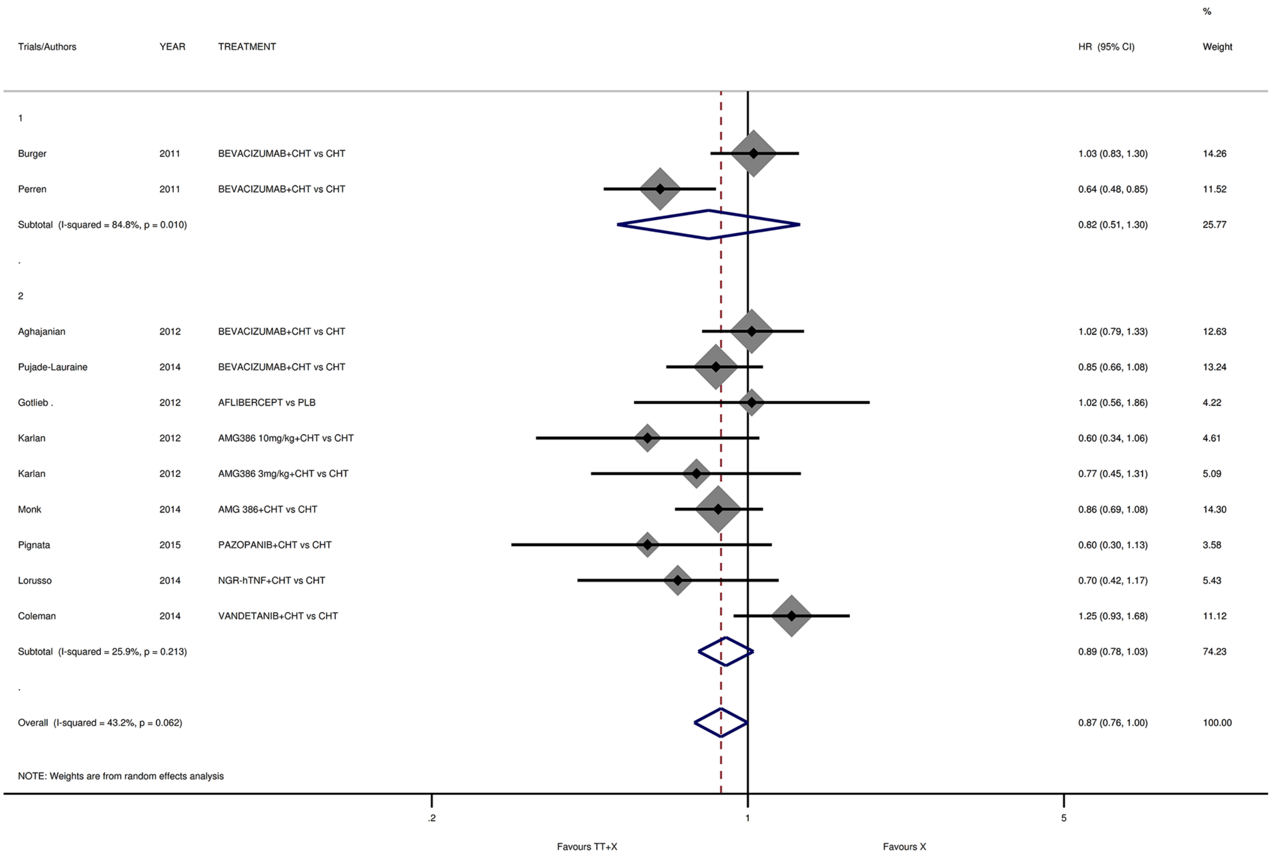

Supplementary Figure S1: Comparison of OS according to line of treatment for anti-angiogenetic agents.

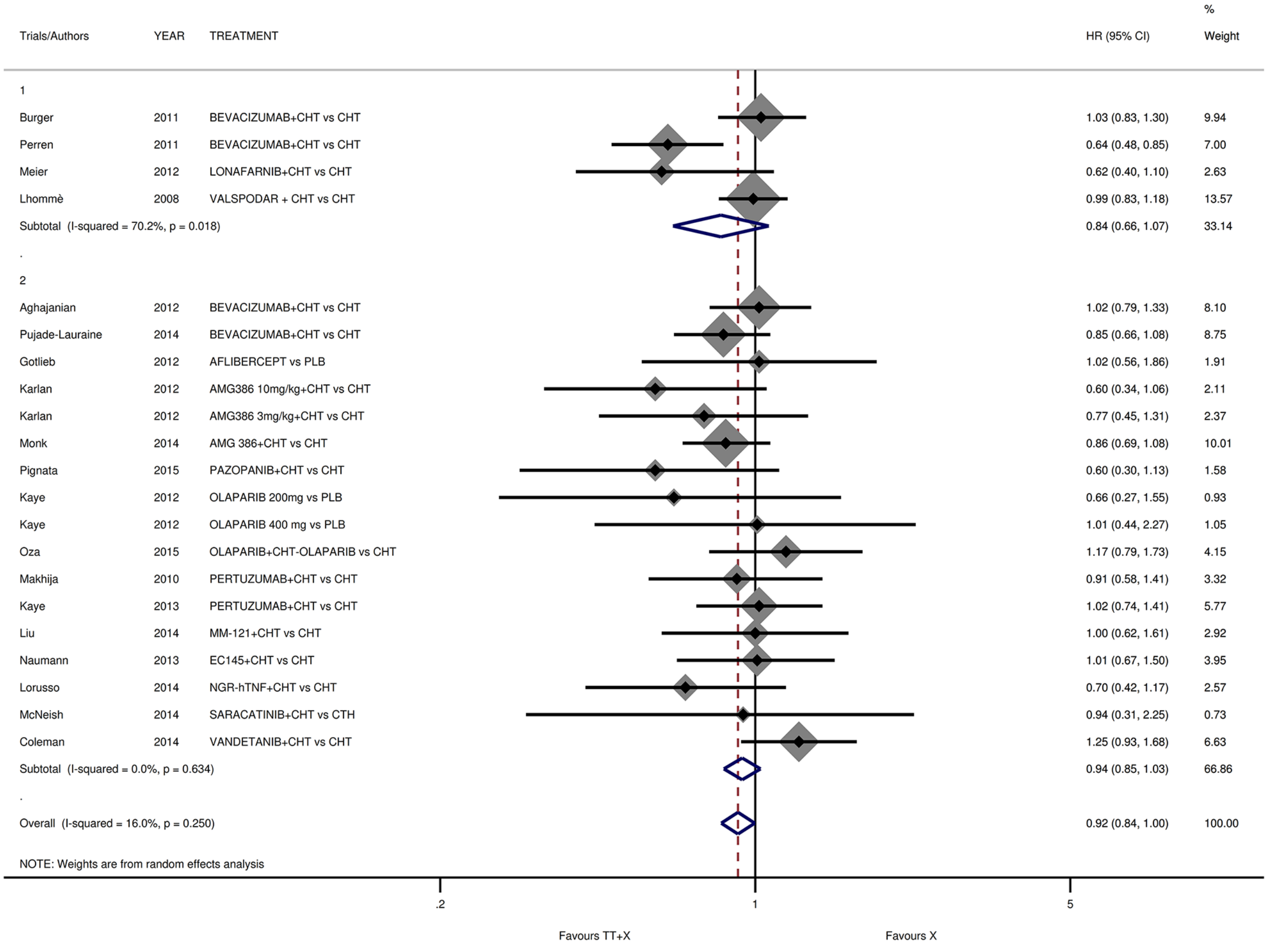

Supplementary Figure S2: Comparison of OS according to line of treatment for all examined pathways.

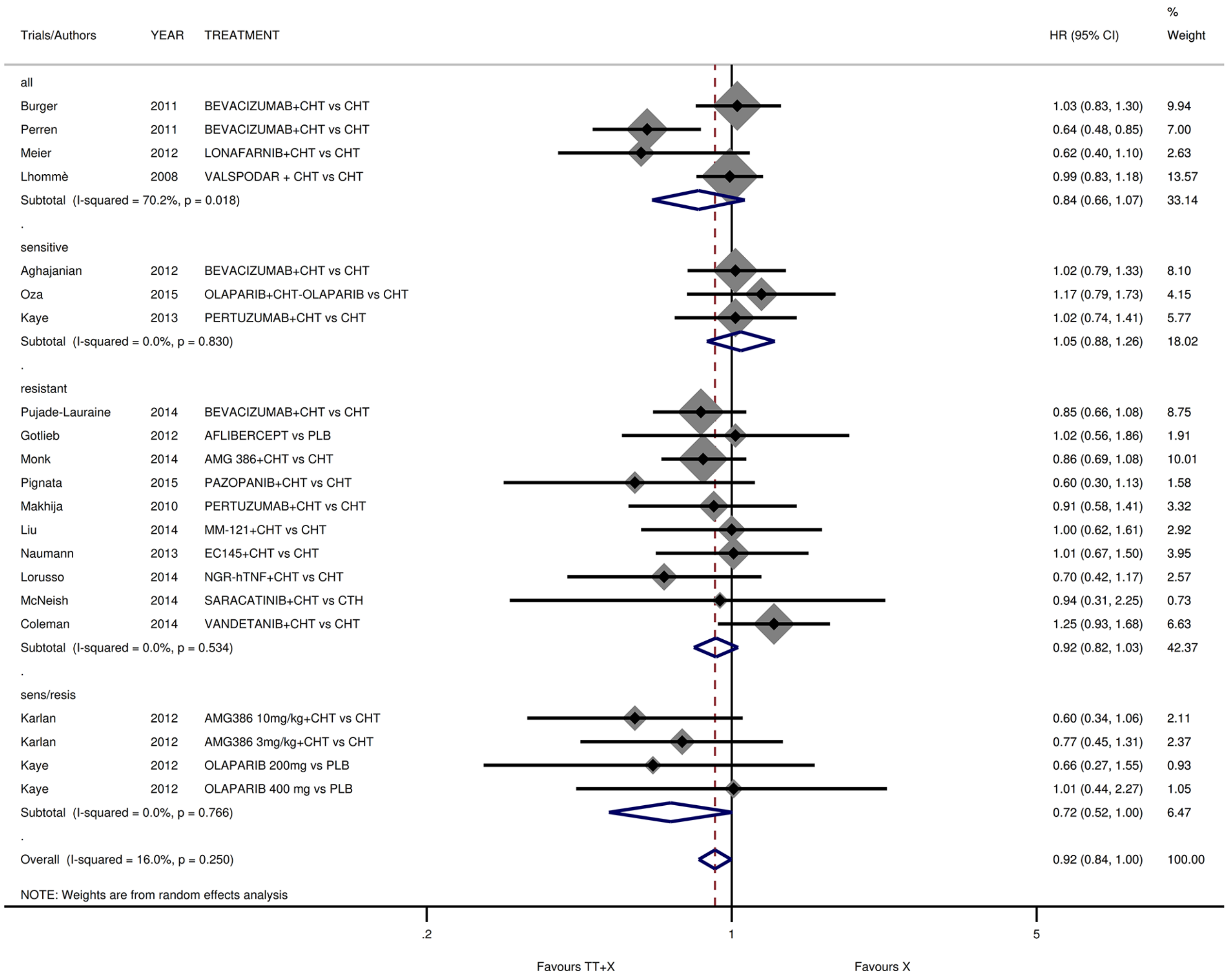

Supplementary Figure S3: Comparison of OS according to platinum status.

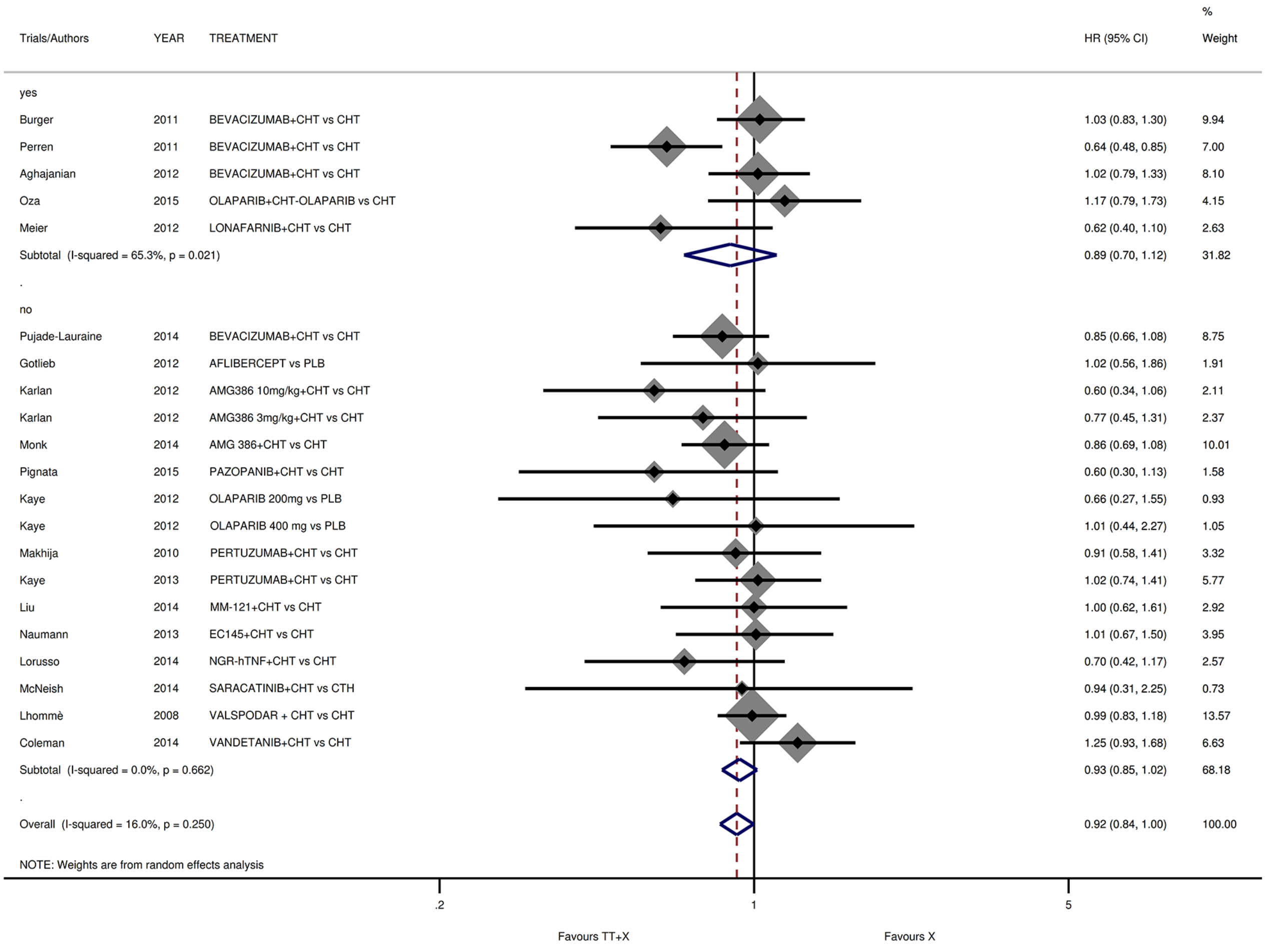

Supplementary Figure S4: Comparison of OS according to maintenance phase.

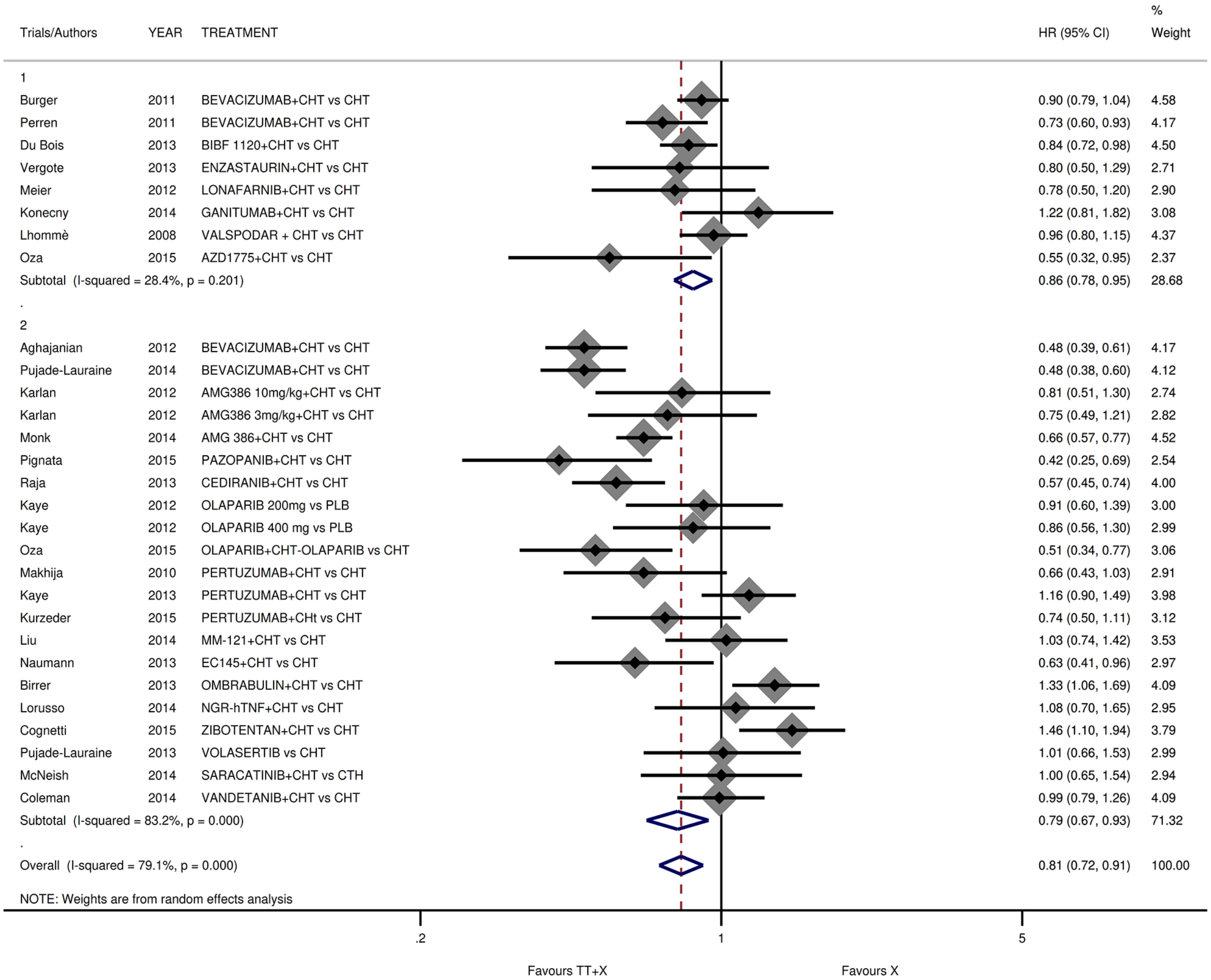

Supplementary Figure S5: Comparison of PFS according to line of treatment for all examined pathways.

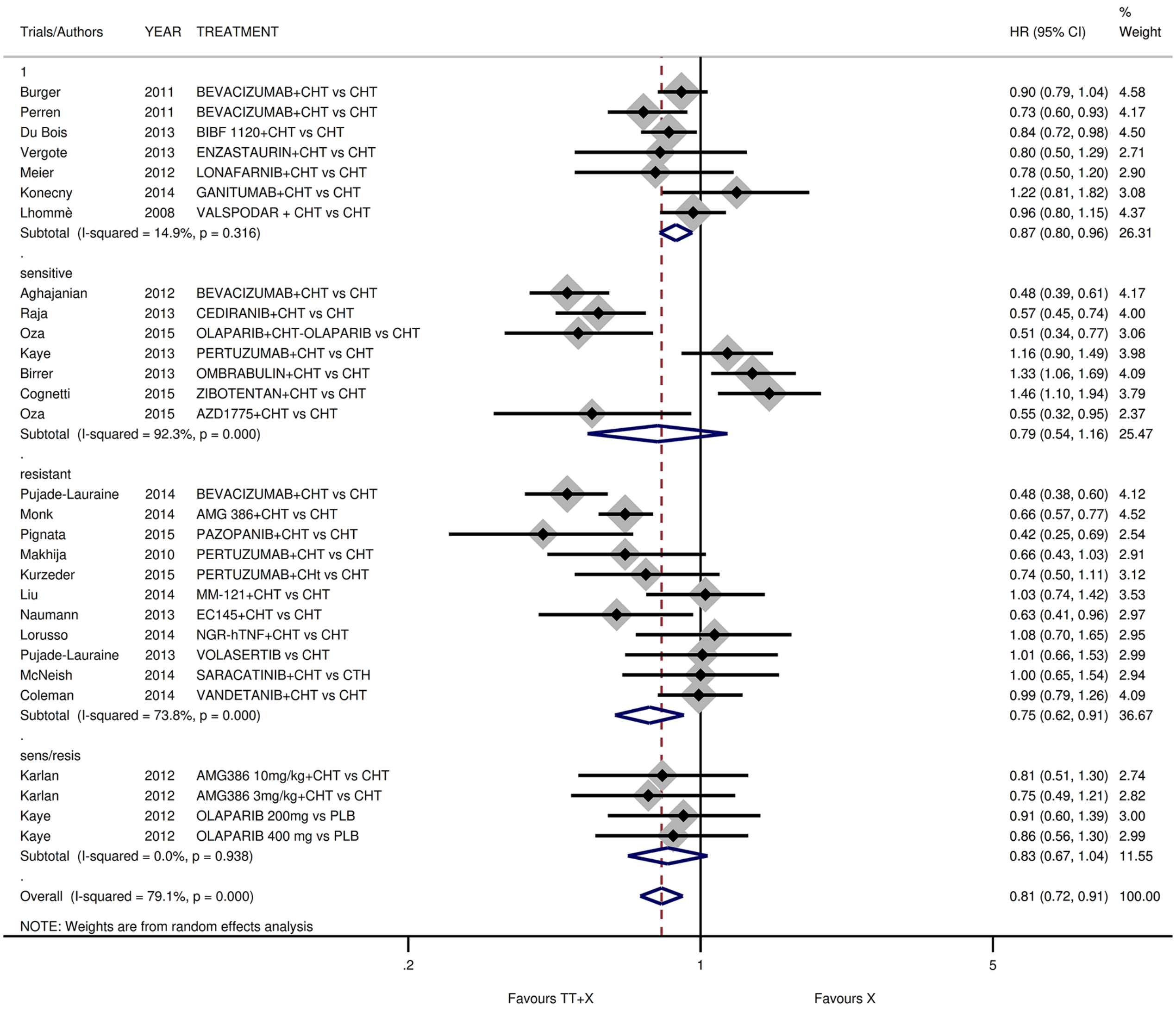

Supplementary Figure S6: Comparison of PFS according to platinum status.

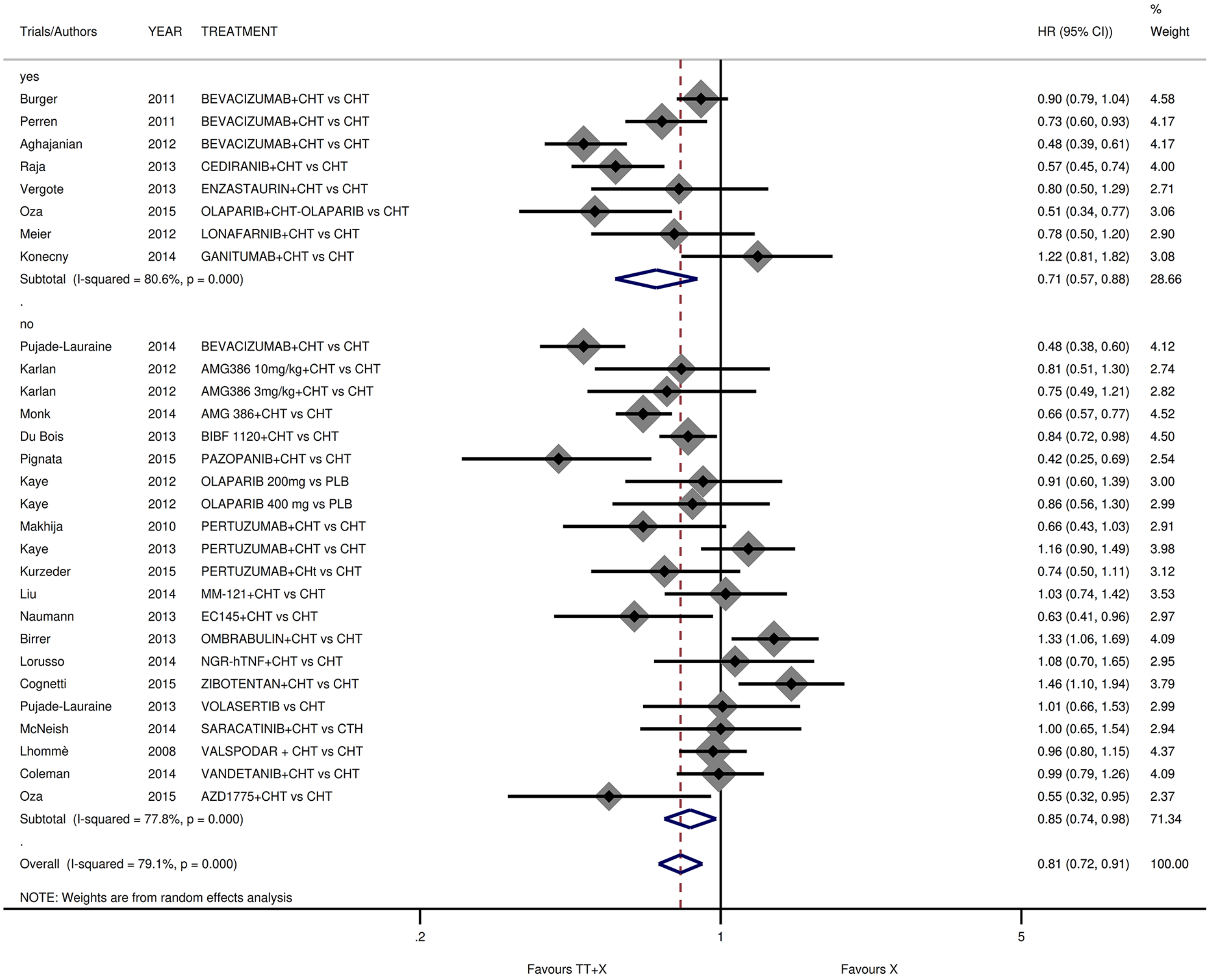

Supplementary Figure S7: Comparison of PFS according to maintenance phase.

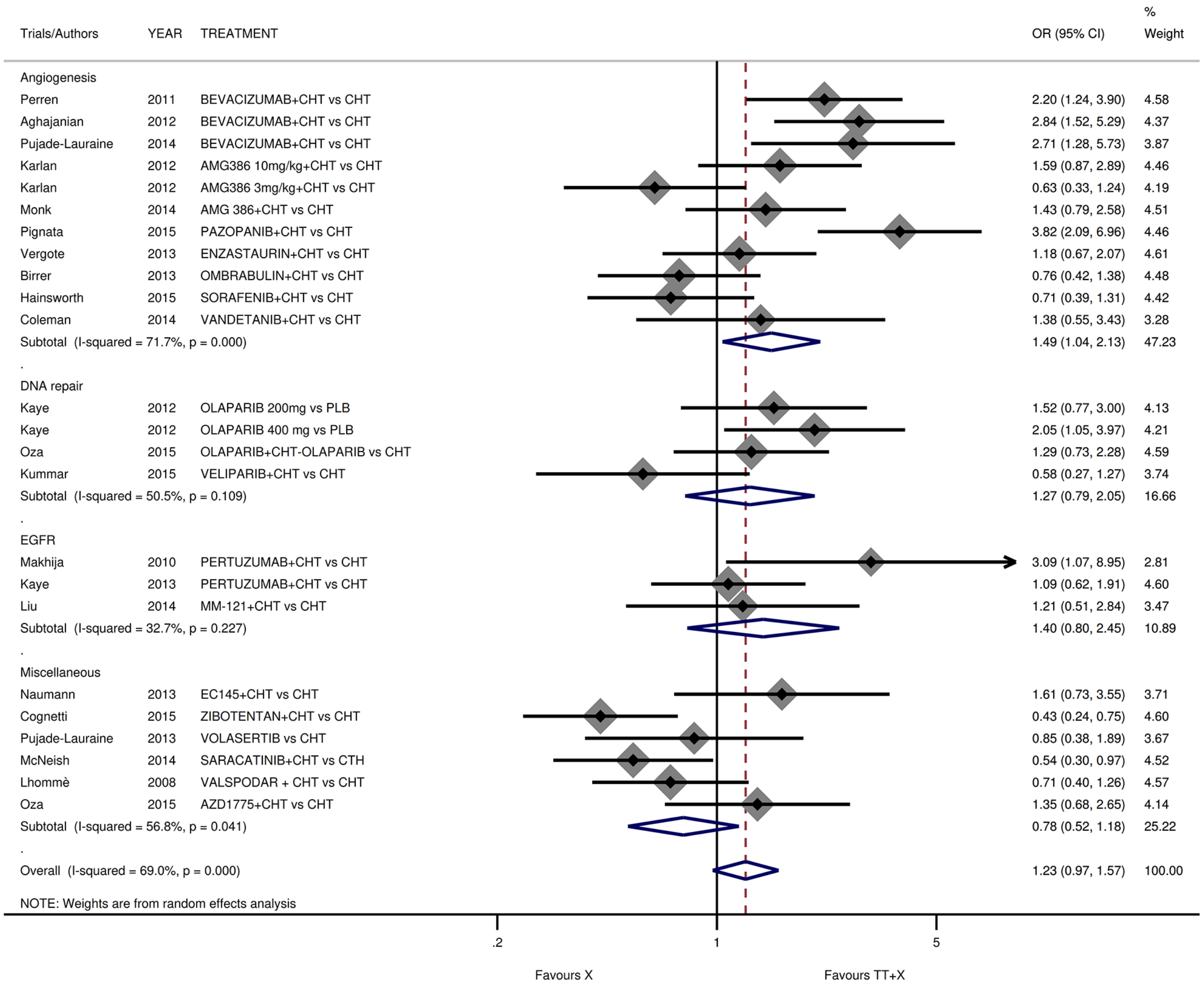

Supplementary Figure S8: Comparison of RR according to each examined pathway.

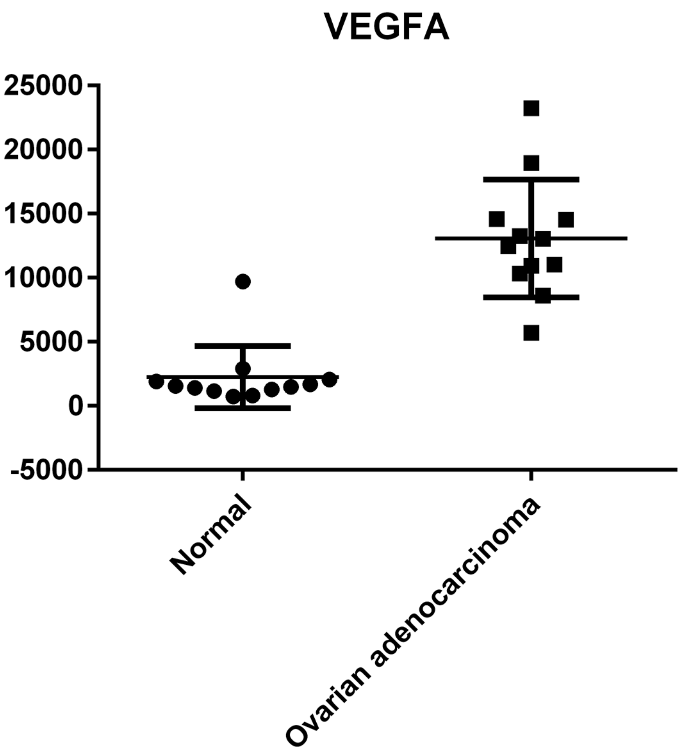

Supplementary Figure S9: Expression levels of VEGFA in EOC dataset.

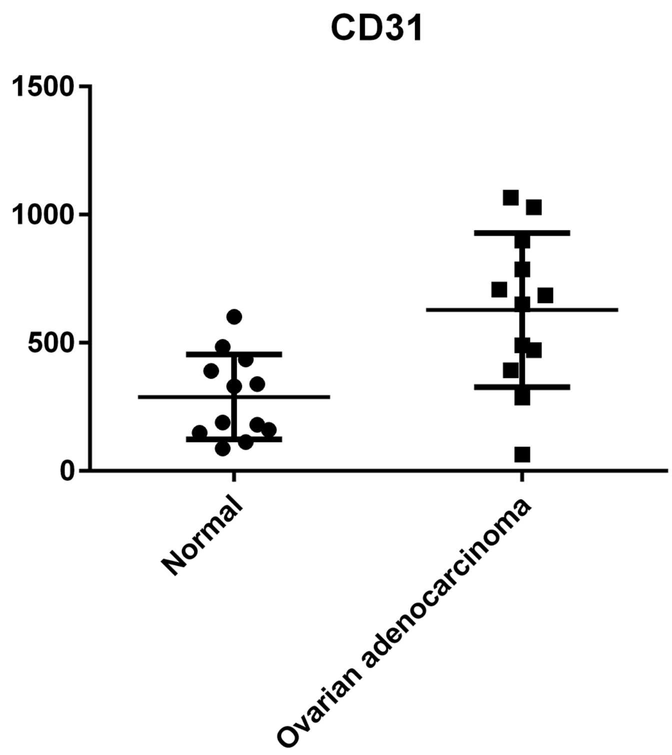

Supplementary Figure S10: Expression levels of CD31 in EOC dataset.

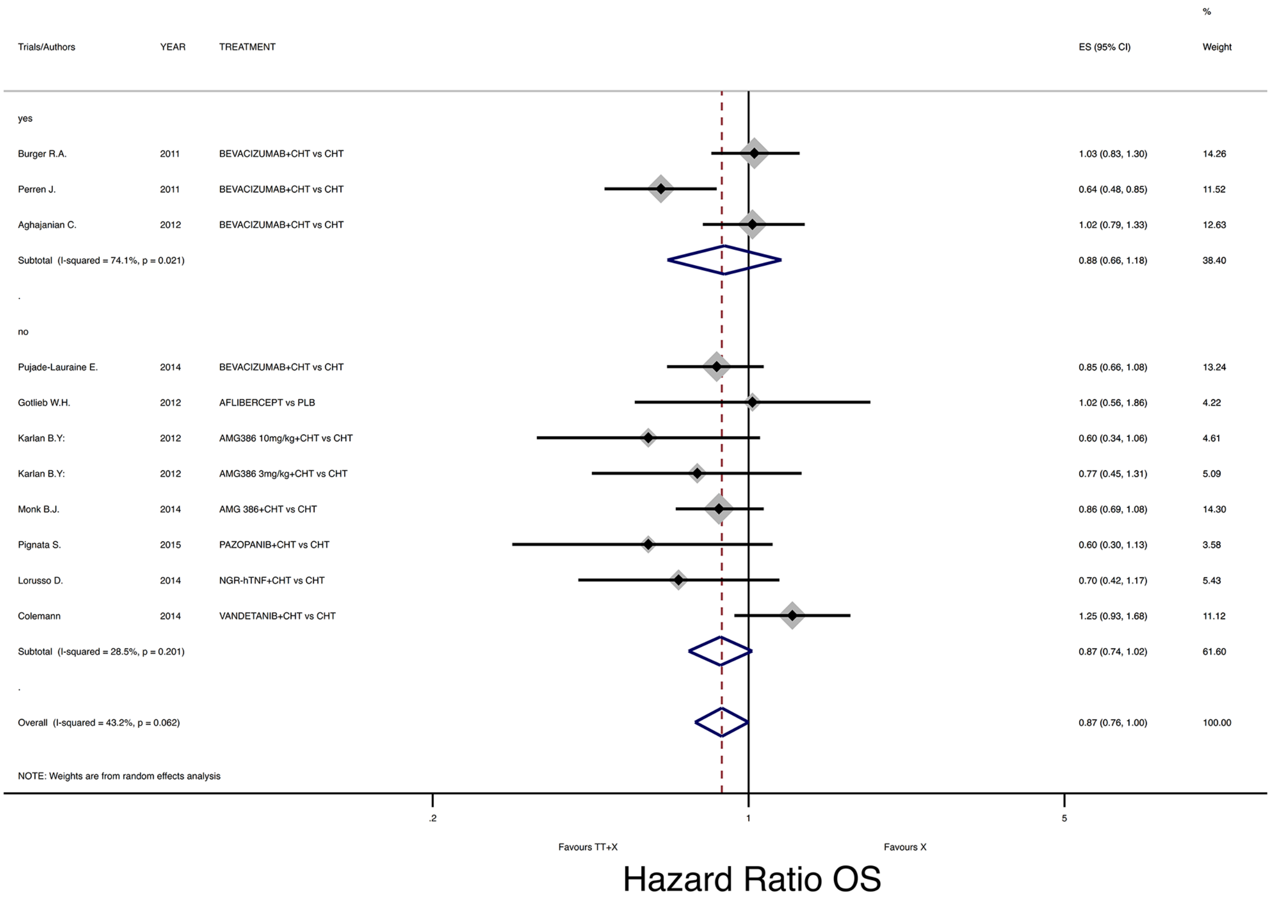

Supplementary Figure S11: Comparison of OS according to manteinance phase for anti-angiogenetic agent.

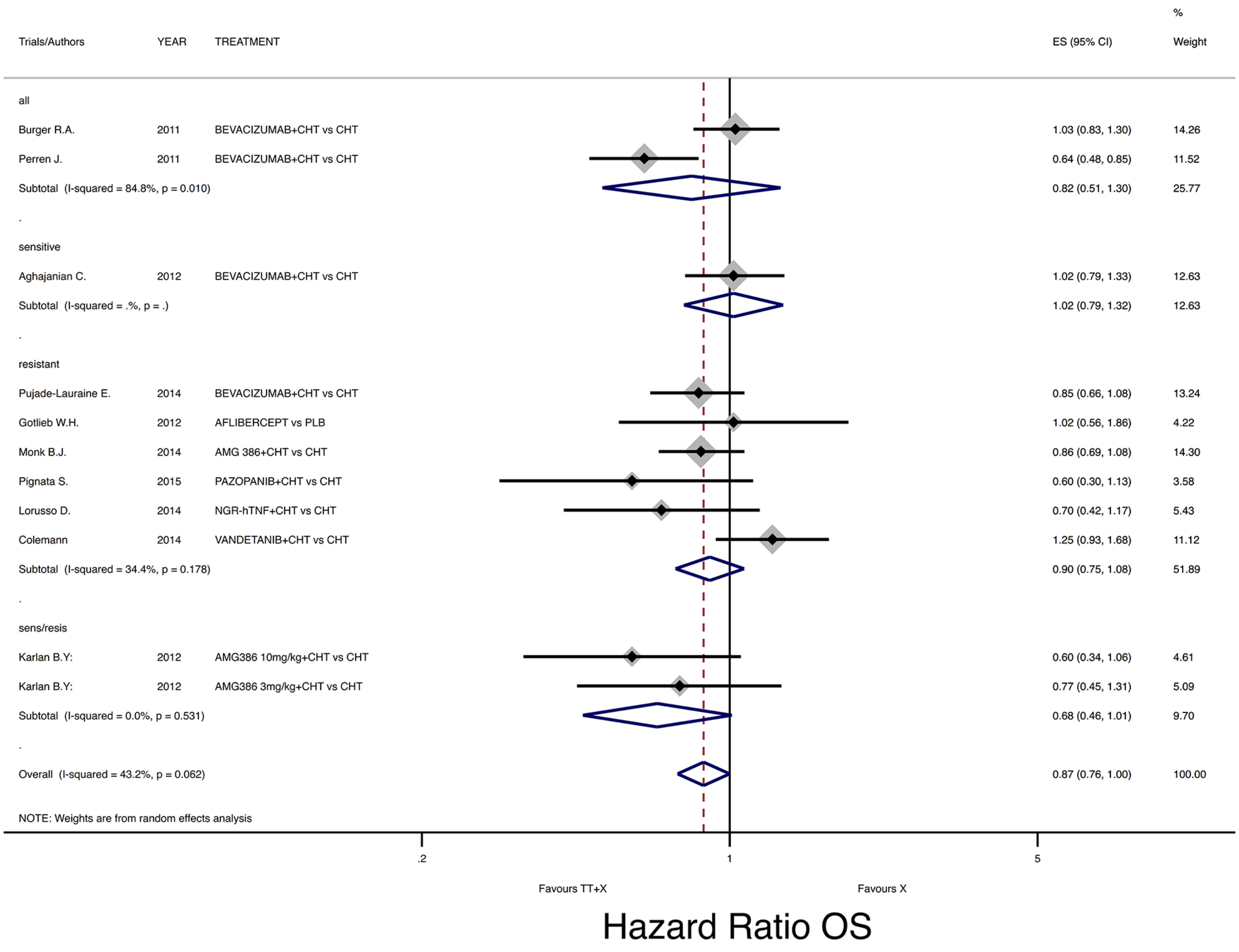

Supplementary Figure S12: Comparison of OS according to platinum status for anti-angiogenetic agent.

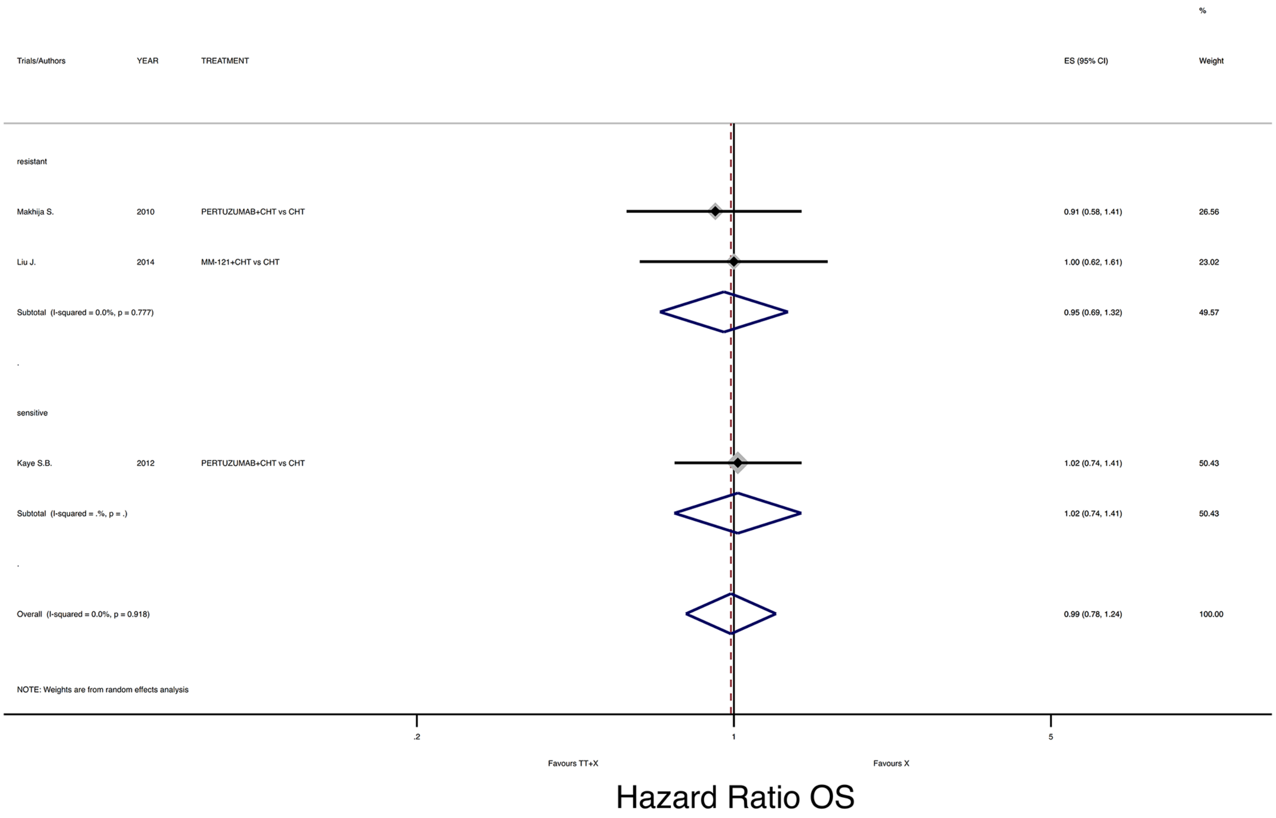

Supplementary Figure S13: Comparison of OS according to platinum status for anti-EGFR agent.

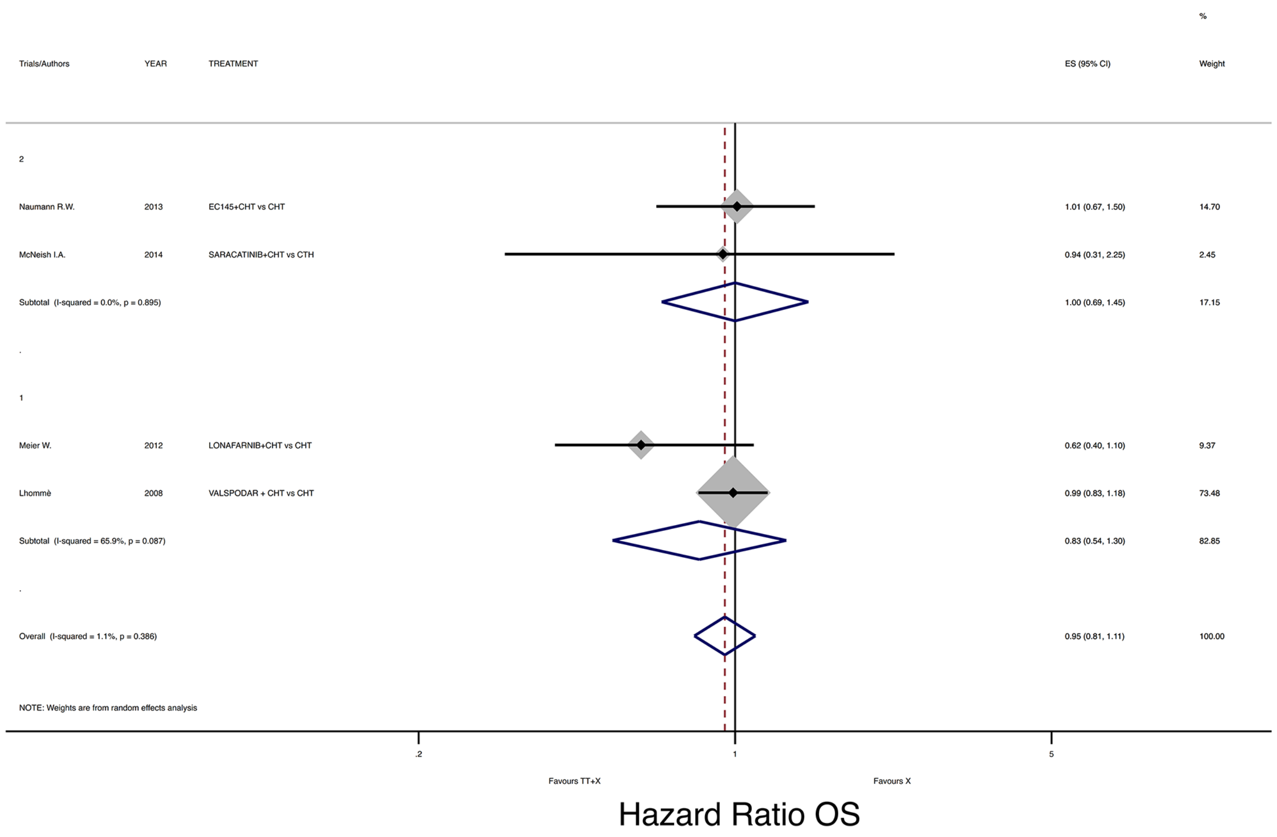

Supplementary Figure S14: Comparison of OS according to line of treatment for miscellaneous.

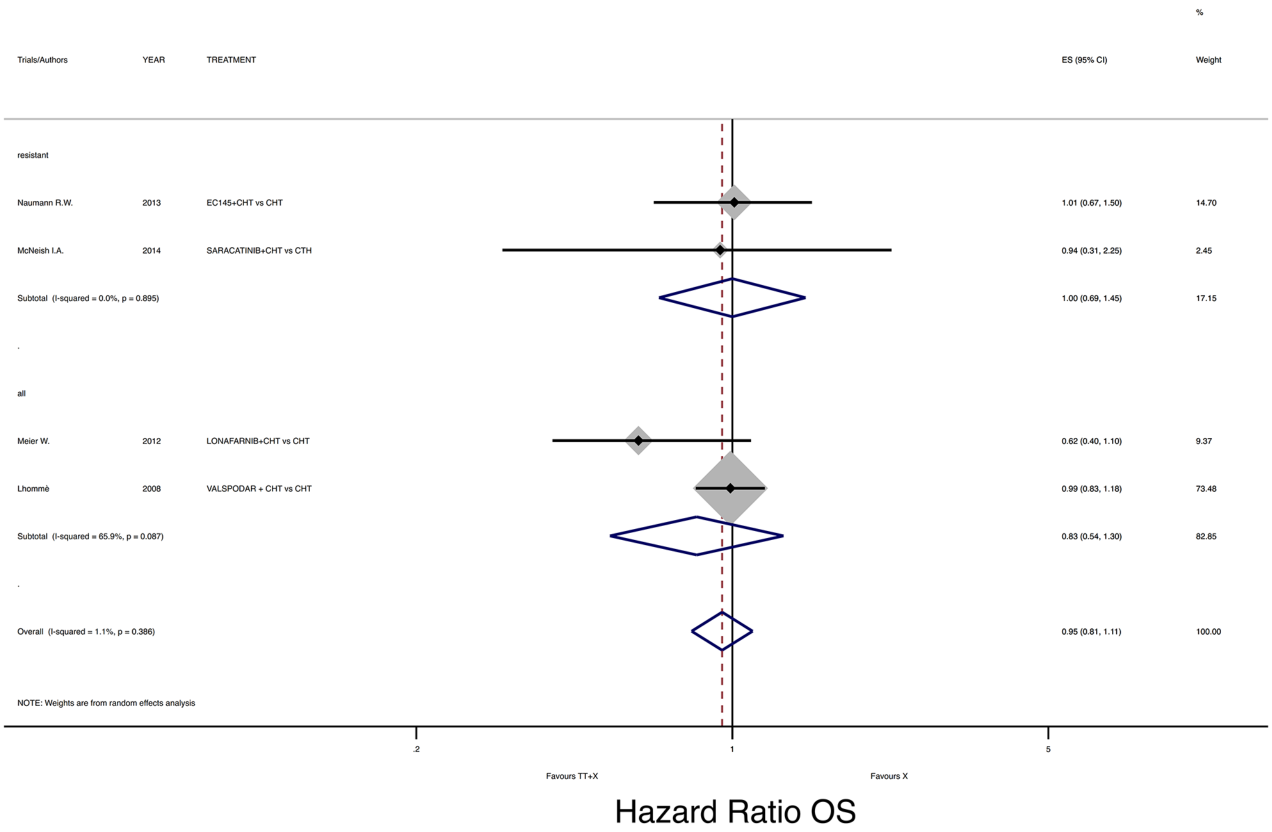

Supplementary Figure S15: Comparison of OS according to platinum status for miscellaneous.

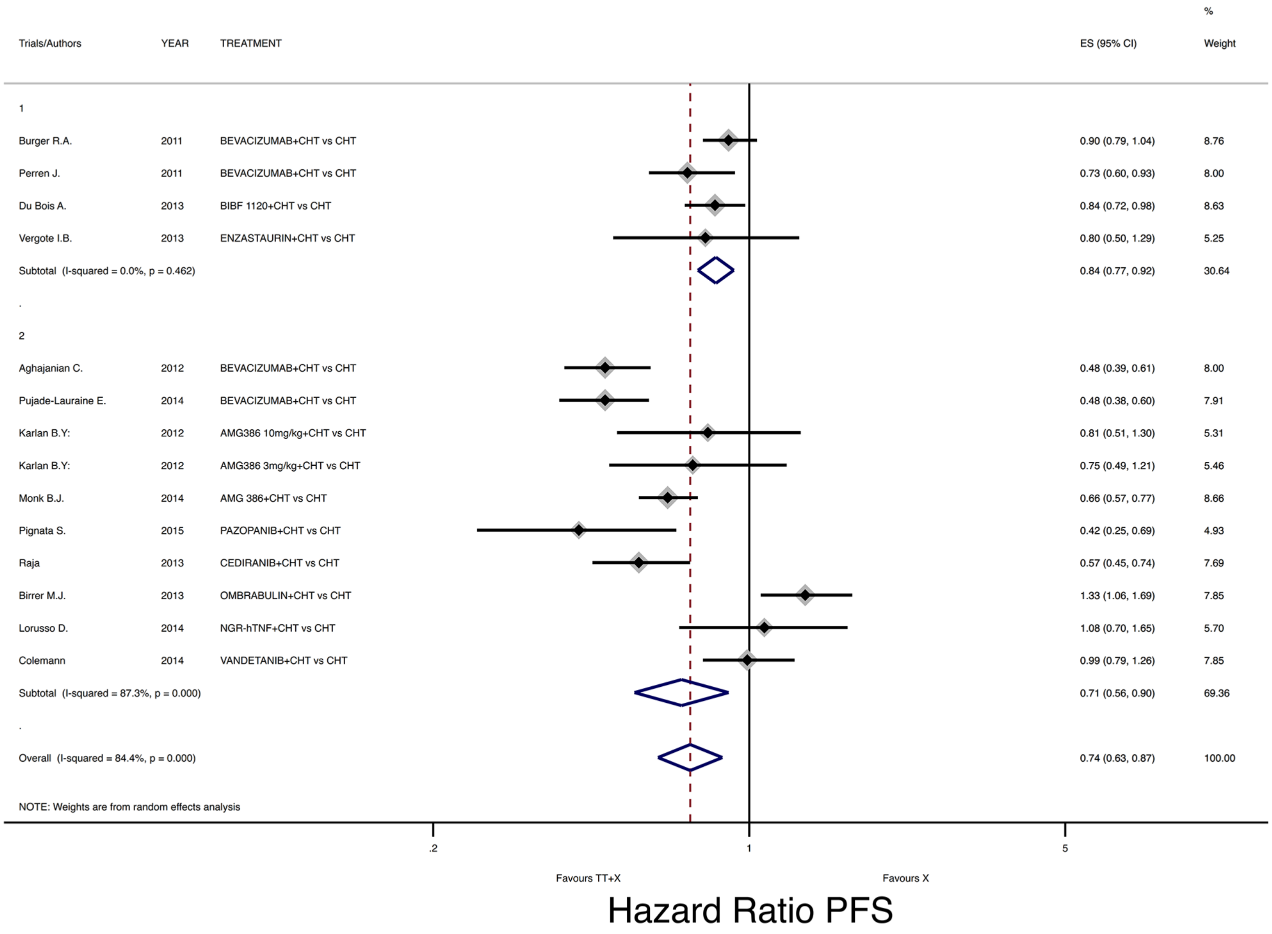

Supplementary Figure S16: Comparison of PFS according to line of treatment for anti-angiogenetic agents.

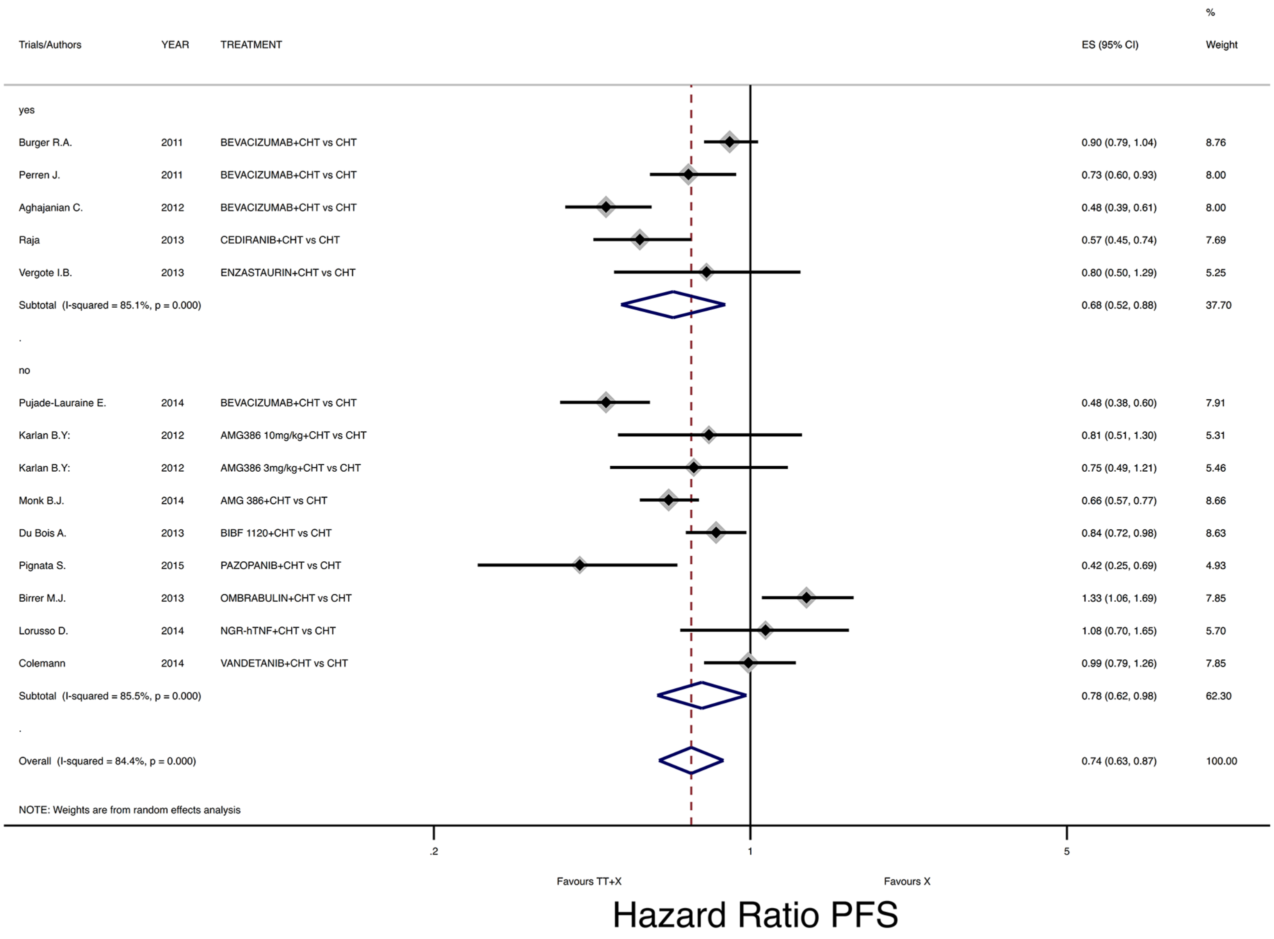

Supplementary Figure S17: Comparison of PFS according to manteinance phase for anti-angiogenetic agents.

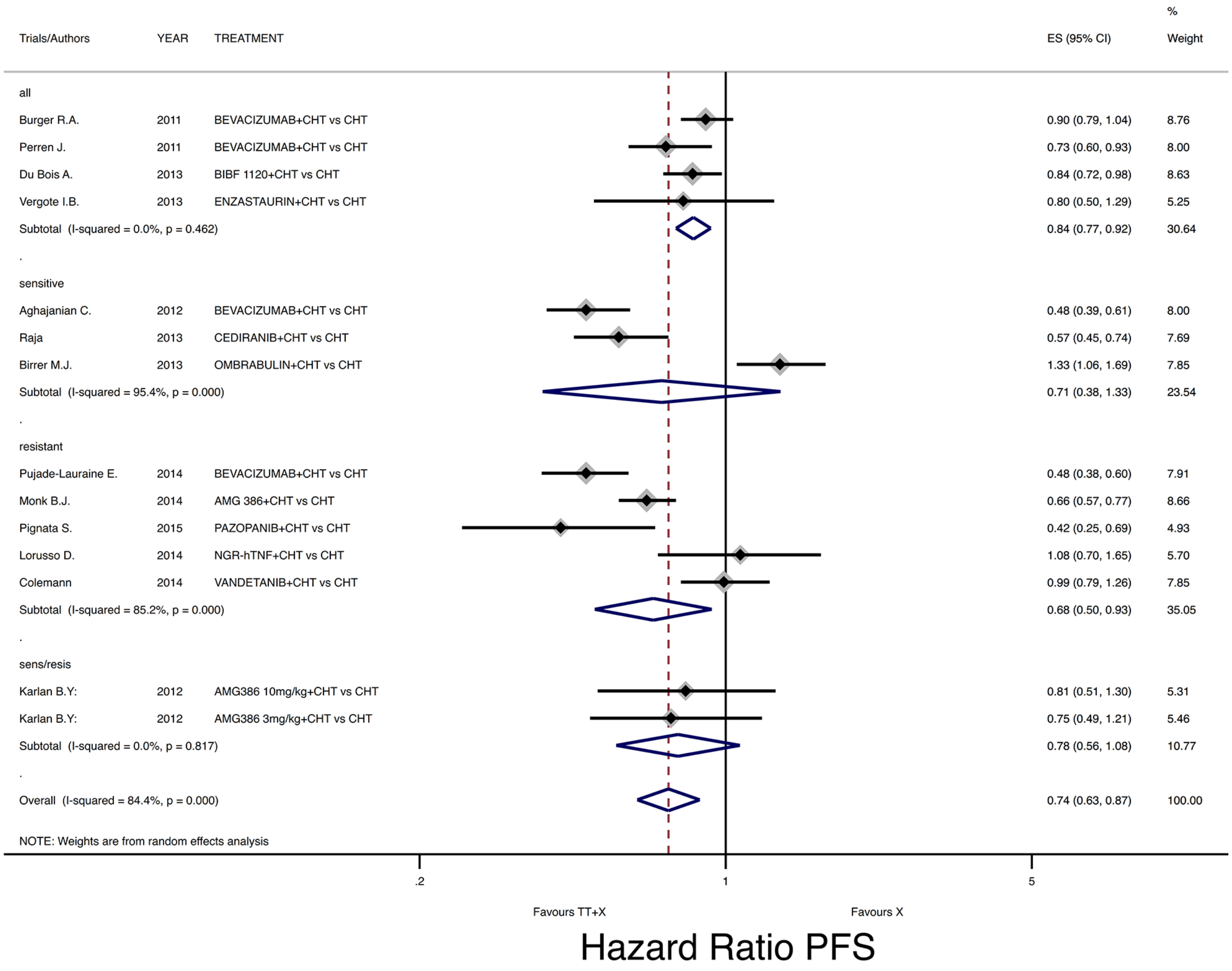

Supplementary Figure S18: Comparison of PFS according to platinum status for anti-angiogenetic agents.

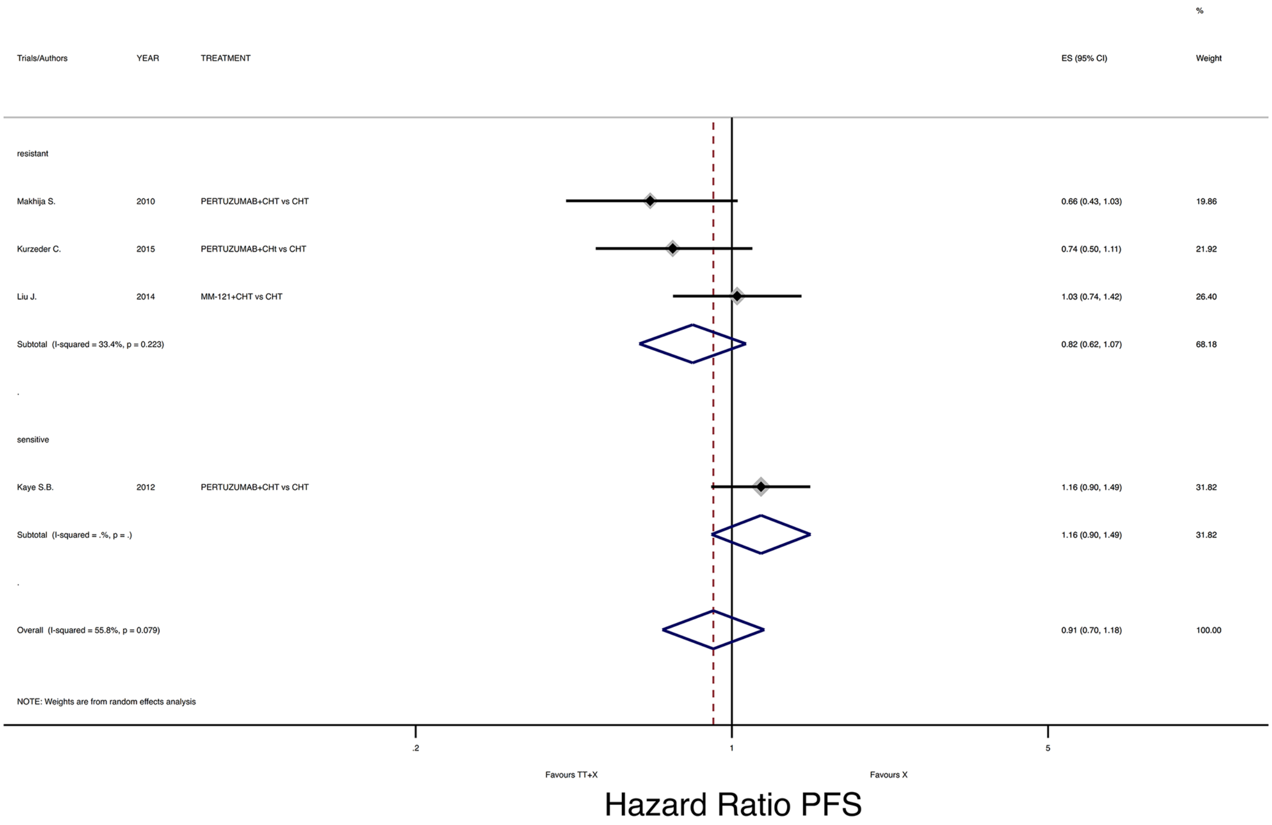

Supplementary Figure S19: Comparison of PFS according to platinum status for anti-EGFR agents.

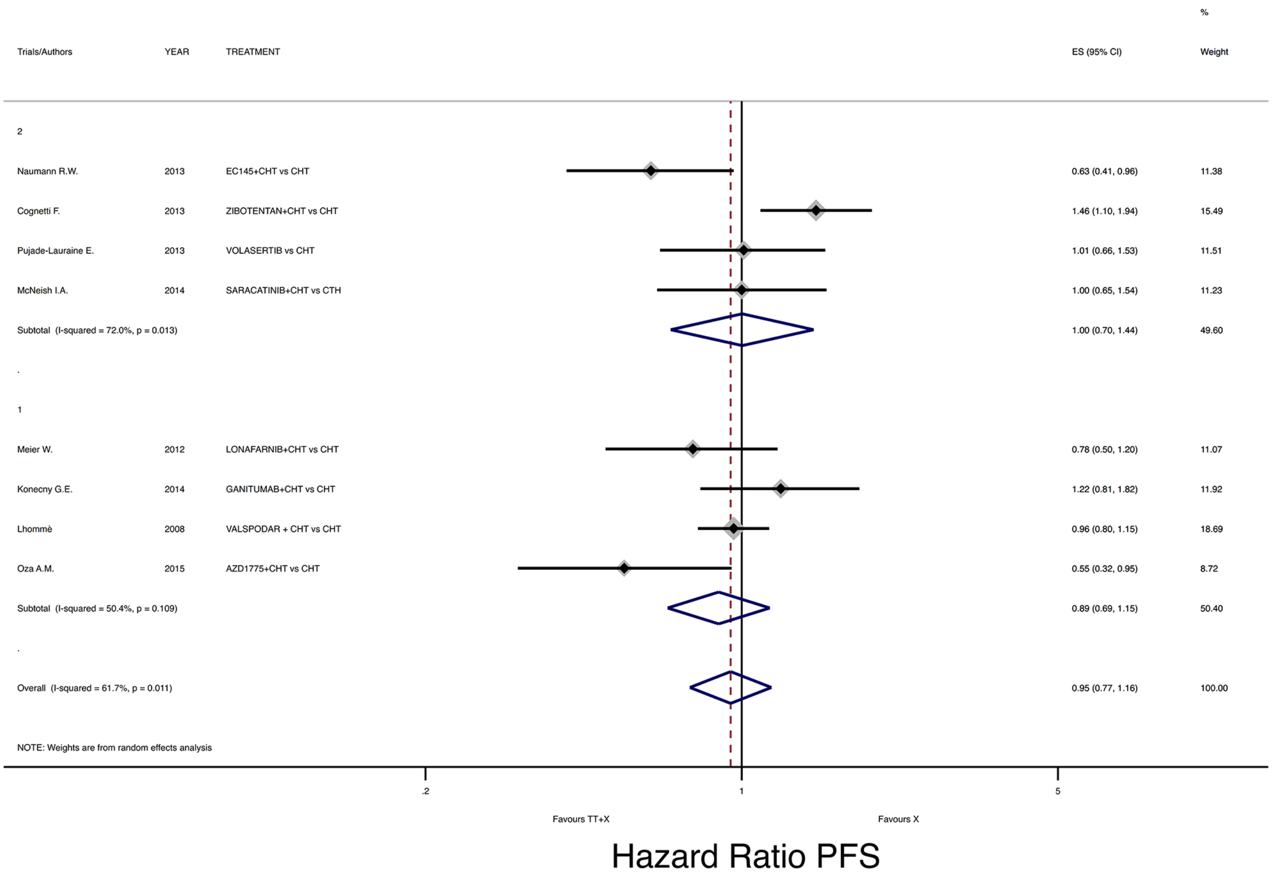

Supplementary Figure S20: Comparison of PFS according to line of treatment for miscellaneous.

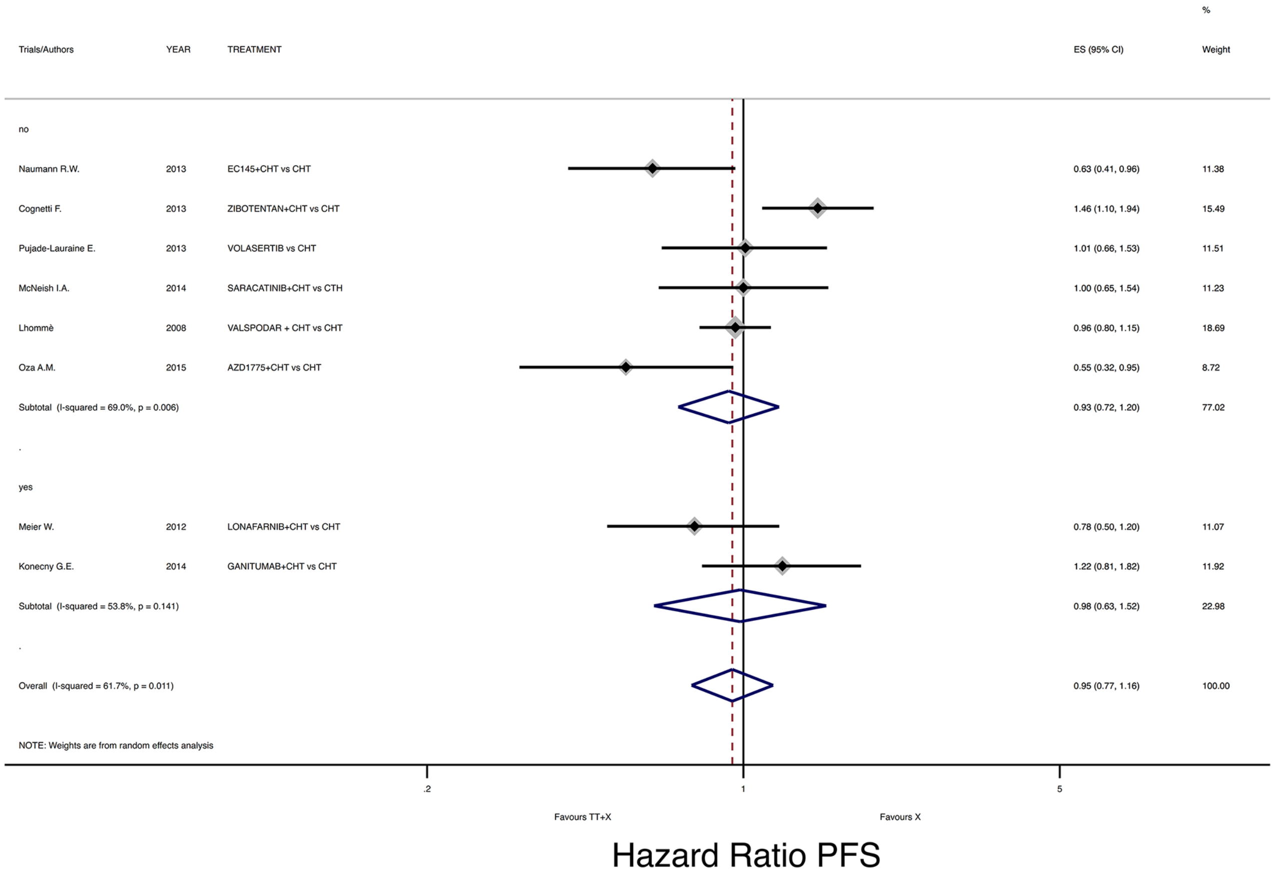

Supplementary Figure S21: Comparison of PFS according to manteinance phase for miscellaneous.

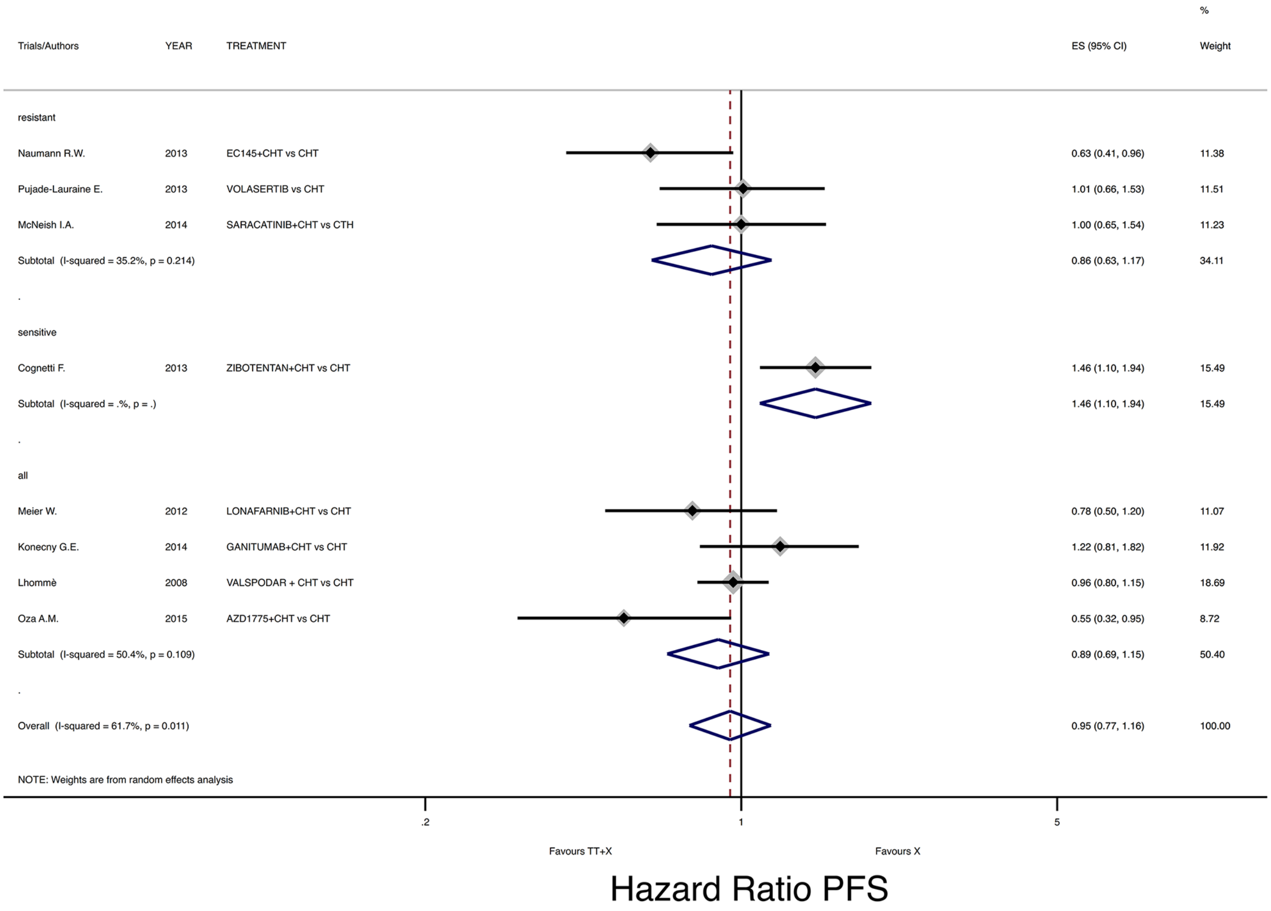

Supplementary Figure S22: Comparison of PFS according to platinum status for miscellaneous.

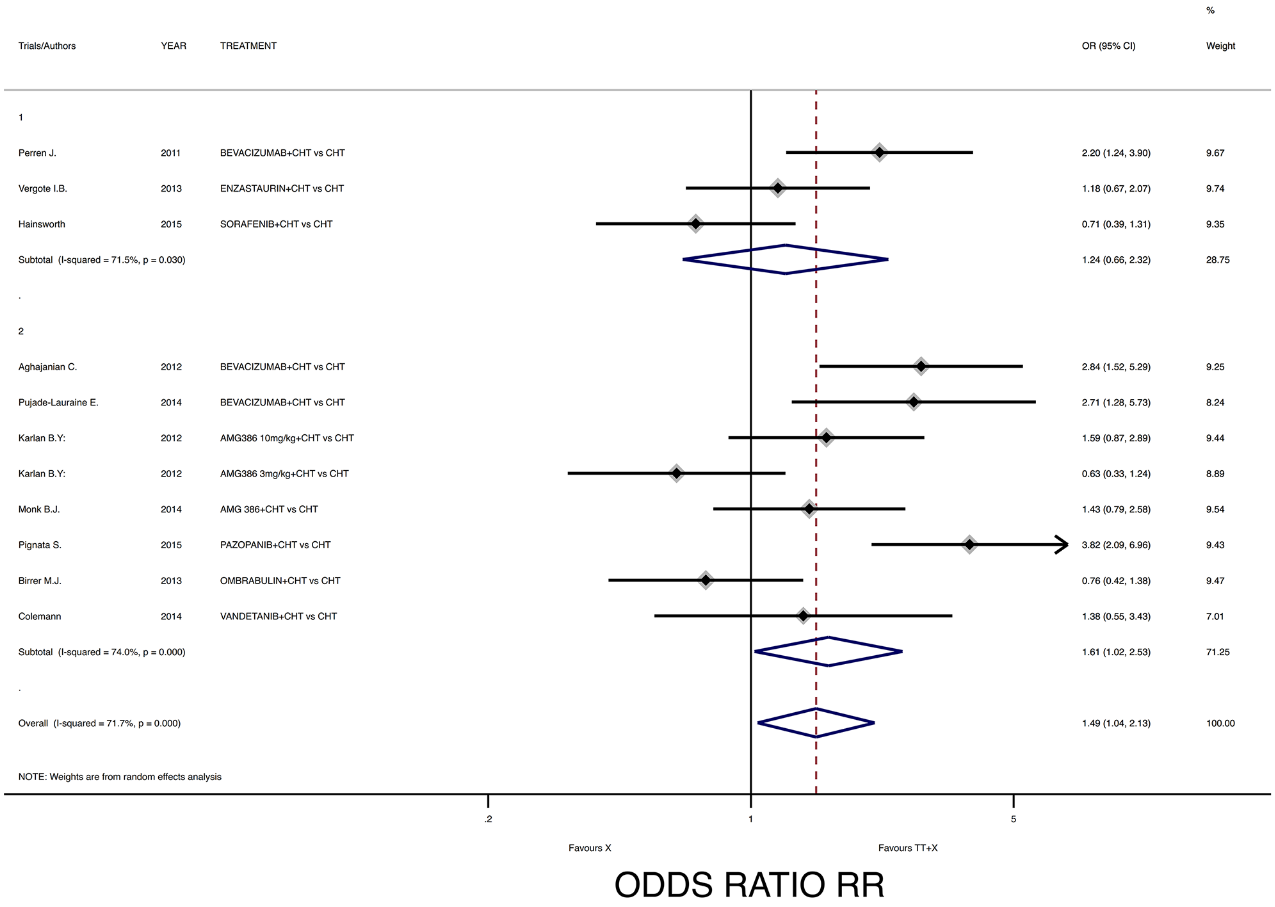

Supplementary Figure S23: Comparison of RR according to line of treatment for anti-angiogenetic agents.

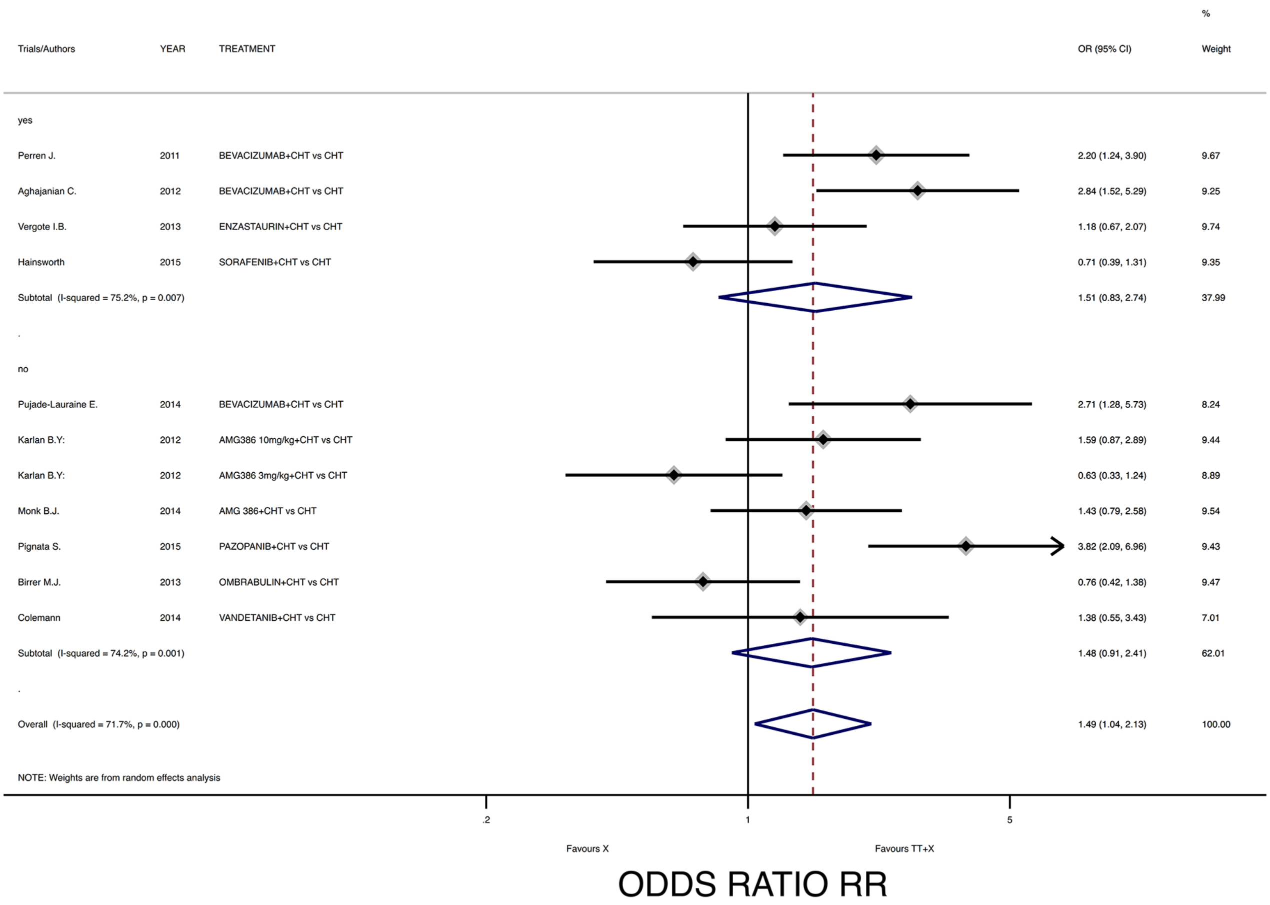

Supplementary Figure S24: Comparison of RR according to manteinance phase for anti-angiogenetic agents.

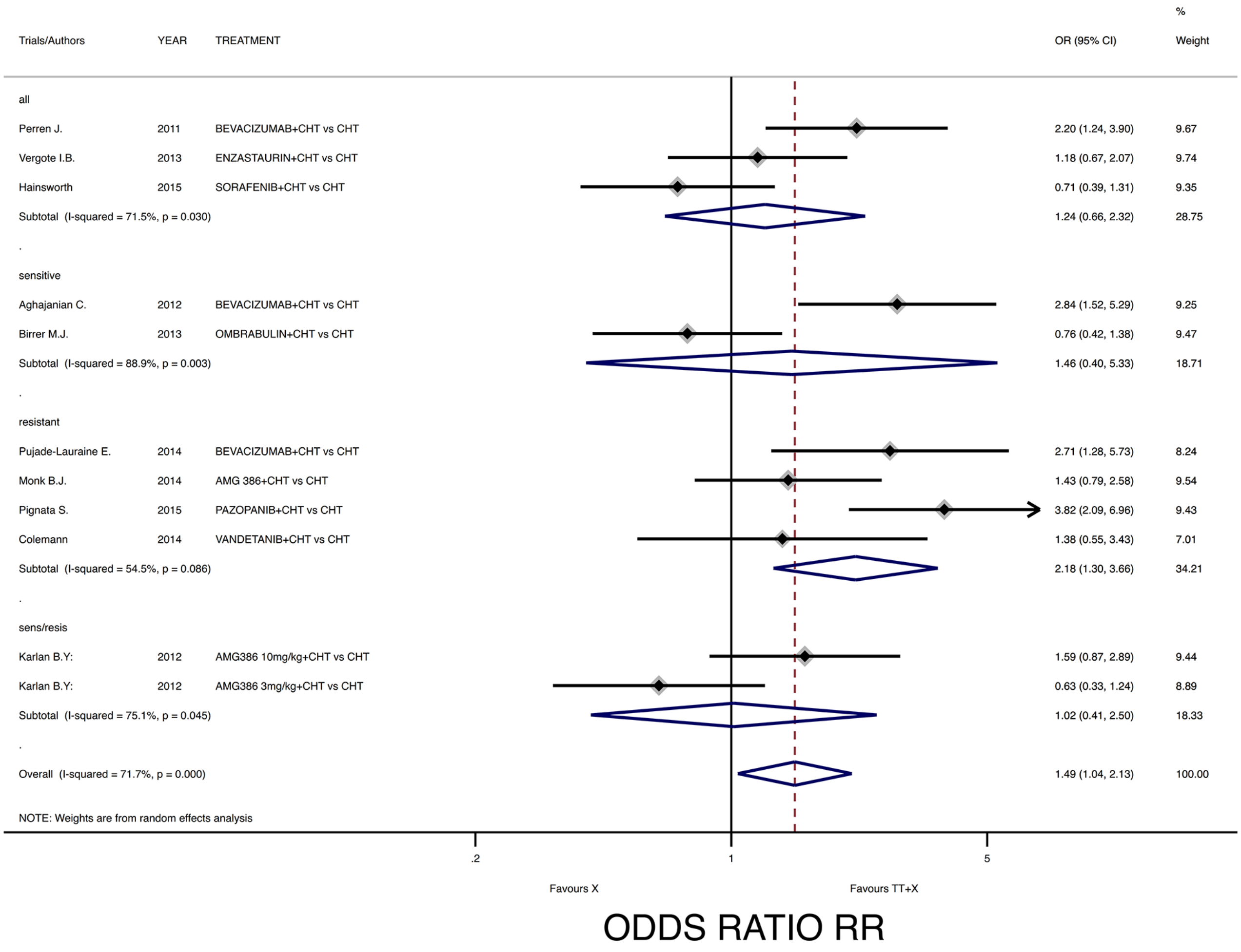

Supplementary Figure S25: Comparison of RR according to platinum status for anti-angiogenetic agents.

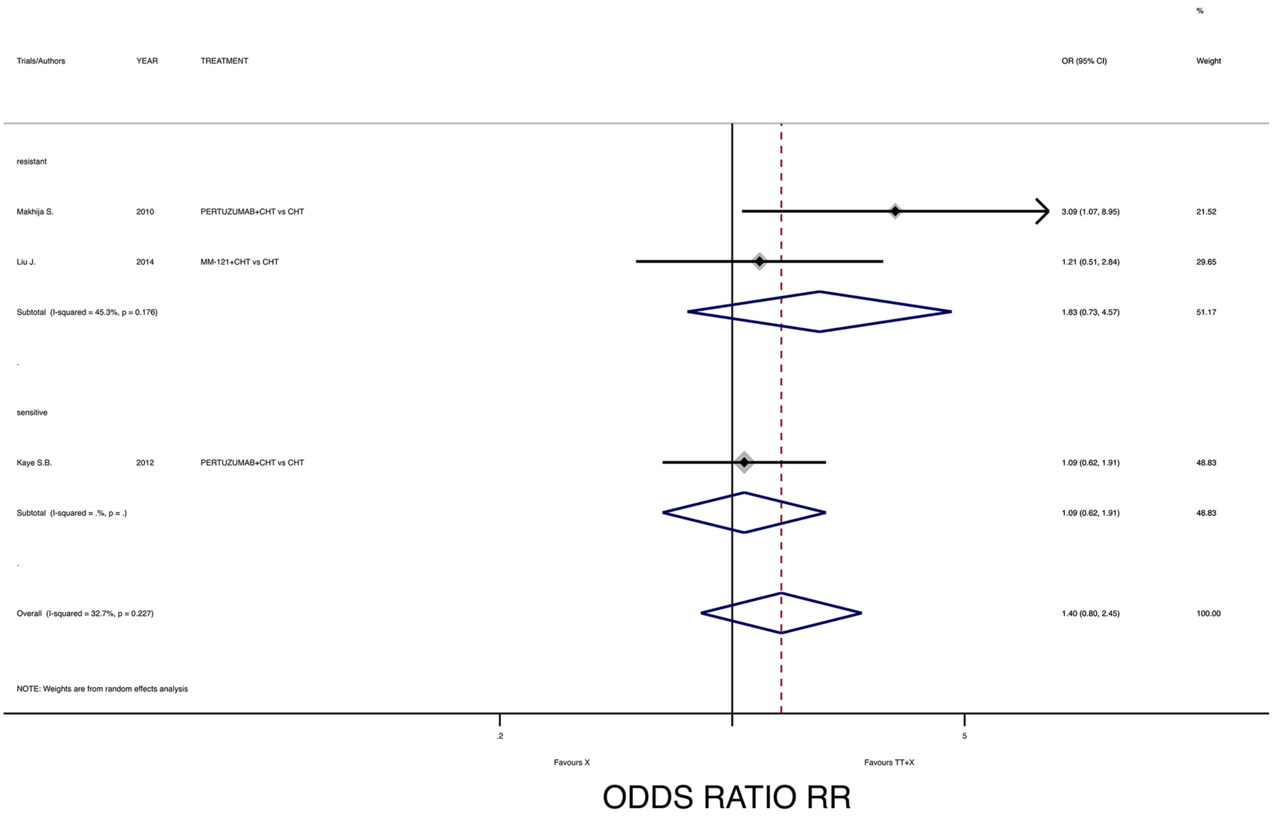

Supplementary Figure S26: Comparison of RR according to platinum status for anti-EGFR agents.

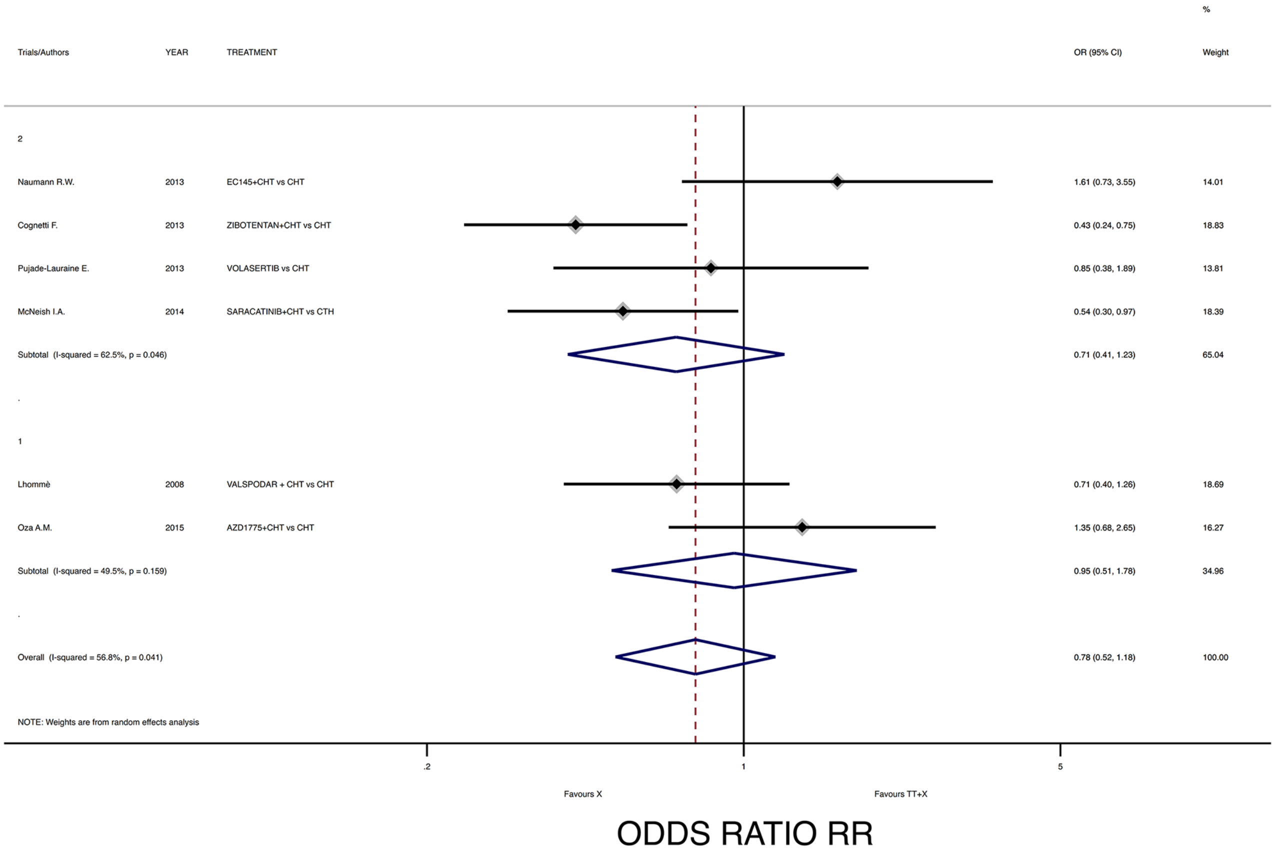

Supplementary Figure S27: Comparison of RR according to line of treatment for miscellaneous.

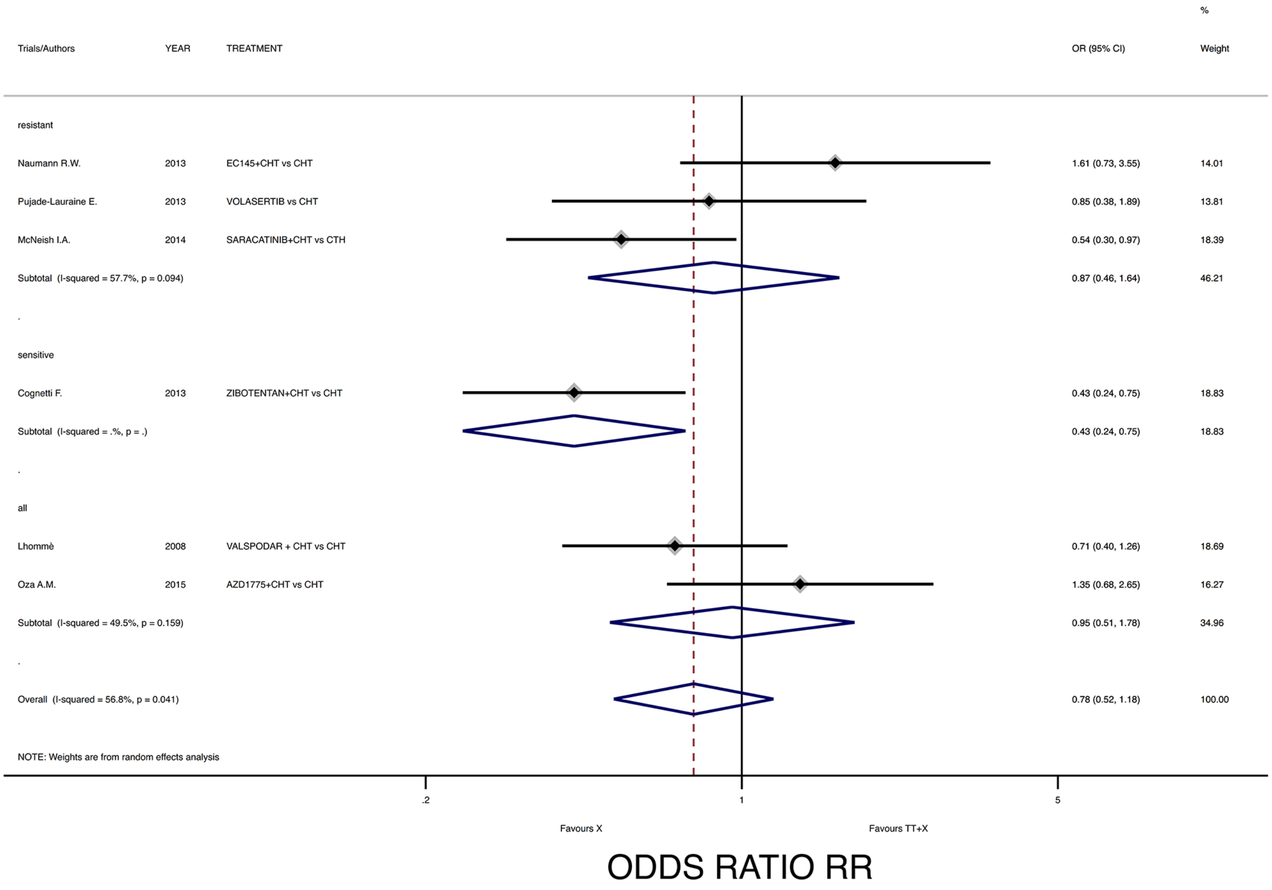

Supplementary Figure S28: Comparison of RR according to platinum status for miscellaneous.
